# Associations between demographics and clinical ideology, beliefs, and practice patterns: a secondary analysis of a survey of randomly sampled United States chiropractors

**DOI:** 10.1186/s12906-023-04225-z

**Published:** 2023-11-09

**Authors:** Zachary A. Cupler, Jordan A. Gliedt, Stephen M. Perle, Aaron A. Puhl, Michael J. Schneider

**Affiliations:** 1 Butler VA Health Care System, Butler, PA USA; 2 Institute for Clinical Research Education, University of Pittsburgh, Pittsburgh, PA USA; 3 Department of Neurosurgery, Medical College of Wisconsin, Milwaukee, WI USA; 4 Big Data Interrogation Group, AECC University College, Bournemouth, Dorset UK; 5 Discipline of Chiropractic, College of Science, Health, Engineering and Education, Murdoch University, Murdoch, WA Australia; 6 Private Practice, Able Body Health Clinic, Lethbridge, AB Canada; 7 Department of Physical Therapy, University of Pittsburgh, Pittsburgh, PA USA; 8 Clinical and Translational Science Institute, University of Pittsburgh, Pittsburgh, PA USA

**Keywords:** Chiropractic, Professional identity, Healthcare systems, Interdisciplinary health teams, Attitude of health personnel, Interprofessional relations

## Abstract

**Background:**

The chiropractic profession in the United States (US) has a long history of intra-professional discourse surrounding ideology and beliefs. Large-scale efforts have evaluated 3 distinctive subgroups of US chiropractors focused on these areas of practice: spine/neuromusculoskeletal, primary care, and vertebral subluxation. To our knowledge, there have not been any prior studies exploring the factors associated with these ideology and belief characteristics of these subgroups. The purpose of this study was to explore, describe, and characterize the association of US chiropractors’ ideology, beliefs, and practice patterns with: 1) chiropractic degree program of graduation, 2) years since completion of chiropractic degree, and 3) US geographic region of primary practice.

**Methods:**

This was a secondary analysis of a cross-sectional survey of a random sample of US licensed chiropractors (*n* = 8975). A 10% random sample was extracted from each of the 50 states and District of Columbia chiropractic regulatory board lists. The survey was conducted between March 2018-January 2020. The survey instrument consisted of 7 items that were developed to elicit these differentiating ideologies, beliefs, and practice patterns: 1) clinical examination/assessment, 2) health conditions treated, 3) role of chiropractors in the healthcare system, 4) the impact of chiropractic adjustments [spinal manipulation] in treating patients with cancer, 5) vaccination attitudes, 6) detection of subluxation on x-ray, and 7) x-ray utilization rates. Multinomial regression was used to analyze associations between these 7 ideology and practice characteristic items from the survey (dependent variables) and the 3 demographic items listed above (independent variables).

**Results:**

Data from 3538 respondents (74.6% male) were collected with an overall response rate of 39.4%. Patterns of responses to the 7 survey items for ideologies, beliefs, and practice characteristics were significantly different based on chiropractic degree program of graduation, years since completion of chiropractic degree, and geographic region of primary practice.

**Conclusions:**

Among US chiropractors, chiropractic program of graduation, years since completion of chiropractic degree, and geographic region of primary practice are associated with variations in clinical ideology, beliefs, and practice patterns. The wide variation and inconsistent beliefs of US chiropractors could result in public confusion and impede interprofessional integration.

**Supplementary Information:**

The online version contains supplementary material available at 10.1186/s12906-023-04225-z.

## Background

As the largest complementary and integrative health profession, chiropractic has engaged in much debate around its professional identity and scope of practice [1]. First proposed by the Institute for Alternative Futures, there is potential for at least 3 professional subgroup identities among the United States (US) chiropractic profession where subgroup membership in these 3 subgroups has been successfully predicted by attitudes, ideologies, and practice behaviors [2, 3]. These 3 distinct subgroups are described as: 1) a subgroup focused on correcting spinal subluxations to free the body’s self-healing capacity; 2) a subgroup focused on spine and neuromusculoskeletal conditions; and 3) a subgroup focused on primary care or specialty care dealing with a range of non-musculoskeletal conditions.

Healthcare teams are optimized with shared goals, consistent beliefs, and congruent ideologies. When these components are inconsistent or incomplete for a healthcare profession, this may create barriers to inter-professional collaboration and integration into team-based healthcare systems [4–8]. These barriers may be heightened by intra-professional divisions about professional identity, beliefs, and ideologies that add to the confusion or misunderstanding of the unique healthcare profession’s roles, skills, expectations, and boundaries within an inter-professional healthcare system [9].

Knowledge about the multiple subgroups within the chiropractic profession remains poorly understood, leaving a heightened risk of stagnancy in care integration and inter-professional collaboration [10]. An enhanced understanding of the chiropractic profession’s different subgroups may improve intra- and inter-professional expectations, relationships, and team-based performance. Thus, exploring the chiropractic profession’s intra-professional characteristics and subcultures may provide important insight into healthcare delivery architecture in the context of multidisciplinary care integration, and the assessment of successful inter-professional teamwork [4].

Despite evidence suggesting the value of chiropractic integration and utilization into mainstream healthcare systems, barriers to full integration and utilization remain [11–16]. One of the key barriers is the inter-professional concern regarding the variability and inconsistency in chiropractic intra-professional beliefs and subcultures [10, 17]. For example, Bussieres et al. found an association between spine radiographic utilization by US chiropractors and the training institution that they attended [18]. Within the broader healthcare delivery system, it is known that clinician (e.g., age, specialty) and practice setting (e.g., location, patient population) characteristics influence practice behavior, which can lead to variation in cost and quality [19, 20]. For example, the medical school that a physician attended has been found to be associated with variation in clinical practice patterns, and years of experience influences emergency room physician practice styles [21, 22]. Similarly, physicians who have been in practice longer may be at risk for providing lower-quality care [23]. Further, physician beliefs and access to resources—but not patients’ beliefs—have been implicated in geographic variation in end of life care [24].

Prior work has established that the professional identity of Canadian and European chiropractors—and Australian chiropractic students—influences their clinical practice characteristics [6, 25, 26]. The evaluation of Canadian chiropractors also found an association between chiropractic degree program of graduation and practice beliefs/behaviors [6]. In Denmark, the chiropractor’s identity has been found to influence the quantity of referrals received from medical physicians [27]. Based upon the results of these international studies, it is important to understand the differences in professional characteristics associated with chiropractors in the US, which is the origin of the chiropractic profession and contains the largest number of practicing chiropractors in the world [28].

Among US chiropractors, several clinician-level factors may influence ideologies, beliefs, and practice patterns that contribute to intra-professional variation and subculture. The objective of this study was to explore and evaluate various factors that might provide explanations for the variation among chiropractic subgroups in the US [2]. This study specifically aimed to describe and characterize the associations between US chiropractors’ ideology, beliefs, and practice patterns with: 1) chiropractic degree program of graduation, 2) years since chiropractic degree completion, and 3) US geographic region of primary practice.

## Methods

### Study design, setting, participants

This study is a secondary analysis of data from a primary multi-stage, cross-sectional survey conducted between March 2018 and January 2020. This survey was conducted from a randomly selected, stratified sample of licensed chiropractors in the US (response rate 39.4%). A full description of the primary survey study methodology and results is described elsewhere [2]. This cross-sectional survey follows the Consensus-Based Checklist for Reporting of Survey Studies (CROSS) [29].

### Institutional review board

The primary survey study was approved by the University of Bridgeport Institutional Review Board (IRB ID: 2017-10-01).

### Variables collected

**Fig. 1:**
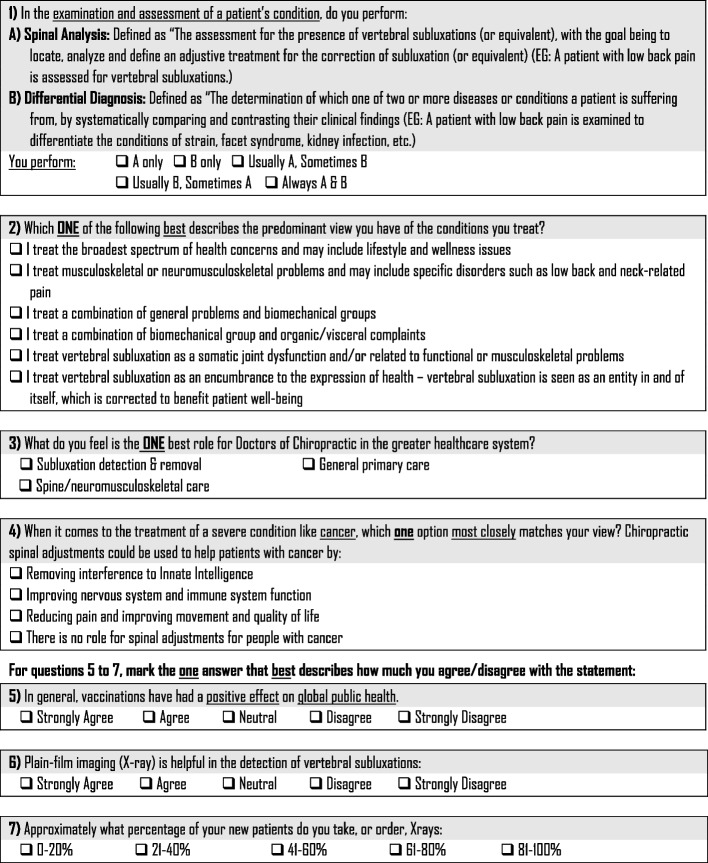
Ideology and practice behavior survey items^2^

Data were collected using a 7-item survey instrument designed to elicit differentiating chiropractic ideologies, beliefs, and practice patterns [2]. The survey instrument was constructed and modeled after similar chiropractic survey analyses conducted in Canada and Europe [6, 25]. Figure 1 is the survey instrument.

Items on the survey instrument solicited ideology, beliefs, and practice pattern information regarding clinical examination/assessment, health conditions treated, role of chiropractors in the healthcare system, the impact of chiropractic adjustments [spinal manipulation] in treating cancer patients, vaccinations attitudes, and x-ray use. Demographic information collected included gender, state or district of primary practice, chiropractic degree program attended, and years since chiropractic program graduation [2].

### Dependent variables

The dependent variables were ideologies, beliefs, and practice patterns, which related to clinical examination/assessment, health conditions treated, role of chiropractors in the healthcare system, the impact of chiropractic adjustments [spinal manipulation] in treating cancer patients, vaccination attitudes, and x-ray use (Fig. 1, Items 1, 2, 3, 4, 5, 6, 7).

### Independent variables

Independent variables included 3 demographics characteristics of the respondents: 1) chiropractic degree program of graduation, 2) years since chiropractic degree program graduation; and 3) region of primary practice location as defined by the US Census Bureau [30].

### Covariates

Covariates included various demographic information, mode of survey completion, and gender. The above-described independent variables served as covariates when not used as the independent variable of interest for the regression model. For example, chiropractic degree program of graduation was the independent variable when years in practice and state of primary practice location were used as covariates for this model. (Note: all covariates were categorical).

### Statistical methods

Statistical analyses were completed on aggregate data that was obtained from the entirety of the survey administration. Descriptive statistics were calculated for the dependent variables to identify the distribution of answers for each of the 7 items of the survey instrument. Descriptive statistics were also calculated for each of the independent variables and demographic covariates. Statistical analysis was completed using STATA version 16 (StataCorp, College Station, TX, USA).

Three multinomial logistic regression models were performed to evaluate the associations between responses to the dependent variable and independent variables, controlling for all demographic covariates. Stacked box plots were performed to visualize the associations for each hypothesis.

### Regression model 1

Null Hypothesis: There are no significant associations between chiropractic degree program of graduation and ideologies, beliefs, and practice patterns.

For our base outcome, we selected Palmer College of Chiropractic as it is had the largest number of respondents and it is the founding institution to offer a chiropractic program in the US. Any chiropractic program with a response rate of less than 10 surveys was omitted. These included: Canadian Memorial Chiropractic College *n* = 7, D’Youville College *n* = 4, and Keiser University *n* = 1. Respondents who indicated attendance at multiple US chiropractic degree programs prior to graduation, “Multiple” (*n* = 21), or chiropractic degree programs not otherwise classified, “Other” (*n* = 21), were also excluded.

### Regression model 2

Null Hypothesis: There are no significant associations between years since chiropractic degree program graduation and ideologies, beliefs, and practice patterns.

Year of chiropractic degree completion was provided by respondents and then converted to years in practice. For our base outcome, we selected ‘1–10 years’ since this represents the subset of most recent graduates.

### Regression model 3

Null Hypothesis: There are no significant associations between primary US region of practice and ideologies, beliefs, and practice patterns.

Only respondents who reported one active state license were included, as those who reported multiple states did not differentiate their primary state of practice. States and the District of Columbia were organized into regions per the US Census Bureau [30]. US territories were not considered for this survey. For our base outcome, we selected ‘Northeast’ as the geographic reference location.

## Results

Across all 50 states and the District of Columbia, there were a total of 3,538 responses collected from a total of 8,975 chiropractors surveyed (39.4% response rate). The overall proportions and distributions of responses to each of the survey items have been previously described [2].

### Demographic characteristics

Demographic characteristics are shown in Table 1, presenting: mode of survey response, gender, chiropractic degree program of graduation, years in practice, and US census region of practice. The majority of respondents completed the mail-delivered mode of survey (80.7%), while a smaller proportion of respondents completed an online (16.9%) or abbreviated postcard mode of the survey (2.5%). Most respondents were male (74.6%). Of chiropractic degree programs of graduation reported by respondents, 17 US institutions were included.

**Table 1:** Demographics of survey respondents (*n* = 3,538)

	n (%)
*Survey Type*	
Mail	2,775 (80.7)
Online	580 (16.9)
Postcard	85 (2.5)
*Gender*	
Male	2,471 (74.6)
Female	840 (25.4)
Multiple	2 (< 0.1)
*Chiropractic Degree Program of Graduation*	
Cleveland College of Chiropractic – Kansas City	146 (4.4)
Cleveland College of Chiropractic – Los Angeles	49 (1.5)
Life University	409 (12.3)
Life University West	92 (2.8)
Logan University	320 (9.6)
National University of Health Sciences	252 (7.6)
New York Chiropractic College	285 (8.6)
Northwestern Health Sciences University	231 (6.9)
Palmer College Of Chiropractic – Iowa	704 (21.2)
Palmer College Of Chiropractic – Florida	40 (1.2)
Palmer College of Chiropractic – West	95 (2.9)
Parker University	163 (4.9)
Southern California University of Health Sciences	188 (5.7)
Sherman College of Chiropractic	69 (2.1)
Texas Chiropractic College	85 (2.6)
University of Bridgeport	41 (1.2)
University of Western States	159 (4.8)
*Years Since Completion of Chiropractic Degree*	
1–10	573 (17.4)
11–20	849 (25.8)
21–30	844 (25.7)
31–40	782 (23.7)
> 40	242 (7.4)
*US Geographic Region of Primary Practice*^a^	
Northeast	683 (20.5)
Midwest	895 (26.8)
South	869 (26.1)
West	889 (26.7)

^a^US Census Region of Practice:

Northeast: Connecticut, Maine, Massachusetts, New Hampshire, New Jersey, New York, Pennsylvania, Rhode Island, Vermont

Midwest: Illinois, Indiana, Iowa, Kansas, Michigan, Minnesota, Missouri, Nebraska, North Dakota, Ohio, South Dakota, Wisconsin

South: Alabama, Arkansas, Delaware, District of Columbia, Florida, Georgia, Kentucky, Louisiana, Maryland, Mississippi, North Carolina, Oklahoma, South Carolina, Tennessee, Texas, Virginia, West Virginia

West: Alaska, Arizona, California, Colorado, Hawaii, Idaho, New Mexico, Montana, Nevada, Oregon, Utah, Washington, Wyoming

The 3 most common chiropractic degree programs of graduation selected by respondents were Palmer College of Chiropractic (Davenport, IA) (21.2%), Life University (Marietta, GA) (12.3%), and Logan University (Chesterfield, MO) (9.6%). Respondents in the second (25.8%), third (25.7%), or fourth (23.7%) decade since graduation approximated a quarter of respondents each. Those respondents in their first decade (17.4%) or fifth decade or more (7.4%) of practice since graduation were less common. The Midwest (26.8%), South (26.1%), and West (26.7%) regions shared similar response rates for primary Census region of practice. The Northeast (20.5%) was less commonly reported as a primary Census region of practice.

Multicollinearity was not observed for any of the models. Multinomial logistic regression models (Table 2) demonstrated statistically significant differences (*P* < 0.001) in proportion of responses to each of the 7 ideologies, beliefs, and practice patterns by chiropractic degree program of graduation (Additional file 1A: Appendix), years since completion of chiropractic degree (by decade) (Additional file 1B: Appendix), and US geographic region of practice (Additional file 1C: Appendix) (Table 2). The 95% confidence intervals for response proportions to each survey item for chiropractic degree program graduation, years since completion of chiropractic degree, and US geographic region of practice are reported in the appendices (Additional file 1A, B, C: Appendix).

**Table 2:** Results of overall model significance analyzing each independent variable with each survey item

Independent Variable	Outcome Variable (Survey Item)	*p*
Chiropractic degree program of graduation	Q1: Scope of Examination	< 0.001
	Q2: Conditions Treated	< 0.001
	Q3: Role in Healthcare System	< 0.001
	Q4: Role of SMT in Cancer Treatment	< 0.001
	Q5: Vaccination	< 0.001
	Q6: Subluxation Detection	< 0.001
	Q7: % of New Patient X-Rays	< 0.001
Year since completion of chiropractic degree	Q1: Scope of Examination	< 0.001
	Q2: Conditions Treated	< 0.001
	Q3: Role in Healthcare System	< 0.001
	Q4: Role of SMT in Cancer Treatment	< 0.001
	Q5: Vaccination	< 0.001
	Q6: Subluxation Detection	< 0.001
	Q7: % of New Patient X-Rays	< 0.001
US geographic region of practice	Q1: Scope of Examination	< 0.001
	Q2: Conditions Treated	< 0.001
	Q3: Role in Healthcare System	< 0.001
	Q4: Role of SMT in Cancer Treatment	< 0.001
	Q5: Vaccination	< 0.001
	Q6: Subluxation Detection	< 0.001
	Q7: % of New Patient X-Rays	< 0.001

*SMT* spinal manipulative therapy

These results indicate that all 3 null hypotheses were rejected. There are significant associations between ideologies, beliefs, and practice patterns and: 1) chiropractic degree program of graduation; 2) years since completion of chiropractic degree; and 3) US geographic region of primary practice.

**Fig. 2:**
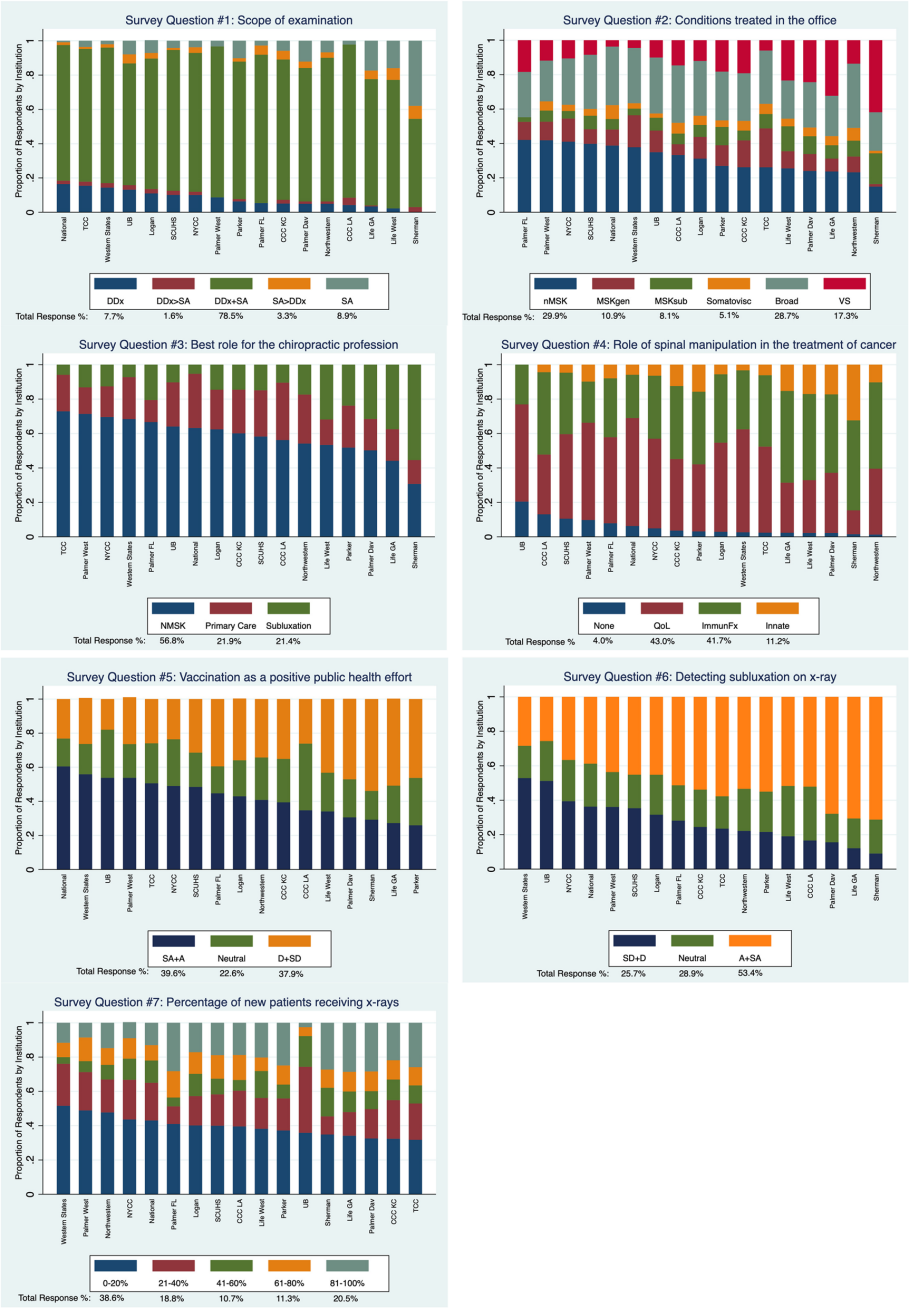
Stacked bar graphs representing association with chiropractic degree program of graduation and ideologies, beliefs, and practice patterns Each bar graph represents the sum of all response by the labeled subgroup on the x-axis and the color, matched to each graphs respective key, is the proportion of respondents within the subgroup who selected each answer and only identified a single correct answer. Respondents who answered for more than once choice were not represented in the bar graphs Palmer: Palmer College of Chiropractic Main Campus, Davenport, IA; CCC-KC: Cleveland University Overland Park, KS; CCC-LA: Cleveland Chiropractic College Los Angles; Life: Life University, GA; Life West: Life Chiropractic College West Hayward, CA; Logan: Logan University, Chesterfield, MO; National: National University of Health Sciences, Lombard IL and Seminole, FL; Northwestern: Northwestern Health Sciences University, Bloomington, MN; NYCC: Northeast College of Health Sciences (formerly New York Chiropractic College), Seneca Falls, NY; Palmer-FL; Palmer College Of Chiropractic Florida Campus, Port Orange, FL; Palmer-West: Palmer College of Chiropractic West Campus, San Jose, CA; Parker: Parker University, Dallas, TX; SCUHS: Southern California University of Health Sciences, Whittier, CA; Sherman: Sherman College of Chiropractic, Spartanburg, SC; TCC: Texas Chiropractic College, Pasadena, TX; Bridgeport: University of Bridgeport, Bridgeport, CT; Western States: University of Western States, Portland, OR Survey Question #1 labels: DDx: Differential Diagnosis only; DDx>SA: Focus on differential diagnosis, sometimes includes spinal analysis; DDx+SA: Equal focus on spinal analysis to detect subluxation and differential diagnosis; SA>DDx: Focus on Spinal analysis, sometimes includes differential diagnosis; SA: Spinal analysis to detect subluxation only Survey Question #2 labels: nMSK: Neuromusculoskeletal Conditions; MSKgen: General and Biomechanical Conditions; MSKsub: Vertebral Subluxation as a Musculoskeletal Condition; Somatovisc: Biomechanical and Organic/Visceral Conditions; Broad: Broad Spectrum of Health Concerns Including Lifestyle and Wellness Issues; VS: Vertebral Subluxation as an Encumbrance to Health Survey Question #3 labels: NMSK: spine and neuromusculoskeletal focused subgroup; Primary Care: General primary care focused subgroup; Subluxation: Subluxation detection and removal subgroup Survey Question #4 labels: None: No Role; QoL: Improving Pain/Quality of Life; ImmuneFx: Improving Nervous System/Immune System Function; Innate: Removing Interference to Innate Intelligence Survey Question #5 labels: SA+A: Strongly Agree and Agree responses; SD+D: Disagree and Strongly Disagree responses Survey Question #6 labels: SD+D: Strongly Disagree and Disagree responses; A+SA: Agree and Strongly Agree responses

Stacked bar graphs demonstrating the proportions of differing responses to the 7 ideologies, beliefs, and practice patterns based on respondents’ chiropractic degree program of graduation are found in Fig. 2. Additional stacked bar graphs for the 7-survey items demonstrating the associations for models 2 and 3, years since chiropractic degree completion (Additional file 2A: Appendix) and primary practice location (US Census region) (Additional file 2B: Appendix), are provided as appendices.

### Association between chiropractic degree program of graduation and ideologies, beliefs, and practice patterns

There was a significant difference in the proportion of responses associated with chiropractic degree program of graduation and all 7 survey items (Fig. 2).

Concerning the scope of examination (survey question 1), the majority (78.5%) of respondents reported that ‘differential diagnosis and spinal analysis were of equal importance’. There were two minority groups, one with a preference for differential diagnosis more than spinal analysis (9.3% responded ‘differential diagnosis only’ and ‘usually differential diagnosis, sometimes spinal analysis’) and one with a preference for spinal analysis more than differential diagnosis (12.2% responded ‘spinal analysis only’ and ‘usually spinal analysis, sometimes differential diagnosis). Respondents from five chiropractic degree programs of graduation (Life GA, Life West, Palmer Dav, Sherman, and UB) selected spinal analysis more than differential diagnosis (range: 12.8–44.8%) in a greater proportion compared to the average total response proportion (12.2%). Respondents from 7 chiropractic degree programs of graduation (Logan, National, NYCC, SCUHS, TCC, UB, and Western States) selected differential diagnosis more than spinal analysis (range: 11.9-18.2%) in a greater proportion to the average total response proportion (9.3%).

Regarding the predominant view of the conditions treated (survey question 2), most respondents selected musculoskeletal and biomechanical conditions (40.8% responded ‘neuromusculoskeletal conditions’ or ‘general biomechanical conditions’). The next largest response was ‘broad spectrum of health conditions’ (28.7%) followed by another subgroup (17.3%) who identified ‘vertebral subluxation as an encumbrance to health’ as the primary conditions treated in their office. The proportion of respondents from six chiropractic degree programs of graduation (CCC KC, Life GA, Life West, Palmer Dav, Parker, and Sherman) who identified ‘vertebral subluxation as an encumbrance to health’ as the primary condition treated was greater (range: 18.2–41.8%) than the average total response proportion (17.3%). Less than 20% of respondents from only one chiropractic degree program of graduation (Sherman) selected musculoskeletal and biomechanical conditions (‘neuromusculoskeletal conditions’ and ‘general and biomechanical conditions’) as the primary conditions treated in the office.

In response to the question about the one best role for the chiropractic profession in the greater healthcare system (survey question 3), the majority (56.8%) of respondents determined the best role for the profession was ‘spine and neuromusculoskeletal focused’. The proportion of respondents from five chiropractic degree programs of graduation (Life GA, Life West, Palmer Dav, Parker, and Sherman) who identified the best role for the chiropractic profession as ‘subluxation detection and removal’ was greater (range: 23.8–55.4%) compared to the average total response proportion (21.4%).

Regarding the role of chiropractic adjustments [spinal manipulation] in treating patients with cancer (survey question 4), most respondents were divided between ‘reducing pain and improving movement and quality of life’ (43.0%) and ‘improving nervous system and immune system function’ (41.7%). A minority of respondents (11.2%) selected ‘removing interference to innate intelligence’. The proportion of respondents from six chiropractic degree programs of graduation (CCC KC, Life GA, Life West, Palmer Dav, Parker, and Sherman) who identified ‘removing interference to innate intelligence’ as the best response to the survey question was greater (range: 12.4–32.3%) compared to the average total response proportion (11.2%).

Concerning vaccination (survey question 5), the respondents were equally divided between agreement (39.6% responded ‘agree’ and ‘strongly agree’) and disagreement (37.9% responded ‘disagree’ and ‘strongly disagree’) with the statement, ‘vaccination is a positive public health effort’. There was a subset of respondents (22.6%) who chose the response ‘neutral’. Respondents from six chiropractic degree programs of graduation (Life GA, Life West, Palmer Dav, Palmer FL, Parker, and Sherman) disagreed (‘disagree’ and ‘strongly disagree’) with the statement, ‘vaccination is a positive public health effort’, in a greater proportion (range: 38.5–55.2%) compared to the average total response proportion (37.9%). Due to the uncertainty about how to interpret the meaning of these ‘neutral’ responses, an upper limit and lower limit for Likert type responses was tabulated. A table was created which presents a lower limit which included only the ‘disagree’ and ‘strongly disagree’ responses, and an upper limit included ‘neutral’ with the ‘disagree’ and ‘strongly disagree’ responses (Table 3).

**Table 3:** Responses to the item ‘In general, vaccinations have had a *positive effect* on *global public health’ (survey question 5).* The first column presents the proportion of respondents who answered ‘Strongly Disagree’ (SD) or ‘Disagree’ (D). The middle column presents the proportion of respondents who answered ‘Neutral’ (N). The third column presents the combination of respondents in the first and second columns (SD + D + N). See Discussion for interpretation of these proportions

Chiropractic degree program of graduation	Proportion of respondents		
	Lower Limit SD + D	N	Upper Limit SD + D + N
Cleveland College of Chiropractic – Kansas City	35.7%	25.2%	60.8%
Cleveland College of Chiropractic – Los Angeles	27.1%	39.6%	66.7%
Life University	51.4%	21.2%	72.6%
Life University West	42.2%	22.2%	64.4%
Logan University	36.0%	21.9%	57.9%
National University of Health Sciences	24.0%	17.1%	41.1%
New York Chiropractic College	23.5%	27.2%	50.7%
Northwestern University of Health Sciences	34.5%	24.8%	59.3%
Palmer College of Chiropractic – Iowa	47.5%	22.7%	70.3%
Palmer College of Chiropractic – Florida	38.5%	17.9%	56.4%
Palmer College of Chiropractic – West	28.0%	19.4%	47.3%
Parker University	46.3%	28.1%	74.4%
Southern California University of Health Sciences	31.3%	20.5%	51.7%
Sherman College of Chiropractic	55.2%	16.4%	71.6%
Texas Chiropractic College	25.3%	24.1%	49.4%
University of Bridgeport	17.9%	28.2%	46.2%
University of Western States	28.4%	16.8%	45.2%
***Average total response proportion***	*37.9%*	*22.6%*	*60.4%*

For the detection of vertebral subluxation on x-ray (survey question 6), the distribution was trimodal. The majority of respondents agreed (53.4% responded ‘agree’ and ‘strongly agree’), while a minority of respondents disagreed (25.7% responded ‘disagree’ and ‘strongly disagree’) or were indifferent (28.9% responded ‘neutral’) with the statement that ‘plain film imaging is helpful in the detection of vertebral subluxations’. Respondents from six chiropractic degree programs of graduation (CCC-KC, Life GA, Palmer Dav, Parker, Sherman, and TCC) agreed (‘agree’ and ‘strongly agree’) with the statement in a greater proportion (range: 53.8–71.2%) compared to the average total response proportion (53.4%). Due to the uncertainty about how to interpret the meaning of these ‘neutral’ responses, an upper limit and lower limit for Likert type responses was tabulated. In similar fashion to survey question 5, a table was created which presents the lower limit (‘agree’ and ‘strongly agree’ responses) and the upper limit (‘agree’, strongly agree’ and ‘neutral’ responses) (Table 4). (*We will discuss the interpretation of these limits in the discussion*.)

**Table 4:** Responses to the item ‘Plain film imaging (x-ray) is helpful in the detection of vertebral subluxations’ (*survey question 6*). The first column presents the proportion of respondents who answered ‘Strongly Agree’ (SA) or ‘Agree’ (A). The middle column presents the proportion of respondents who answered ‘Neutral’ (N). The third column presents the combination of respondents in the first and second columns (SA + A + N). See Discussion for interpretation of these proportions

Chiropractic degree program of graduation	Proportion of respondents		
	Lower Limit SA + A	N	Upper Limit SA + A + N
Cleveland College of Chiropractic – Kansas City	53.8%	21.7%	75.5%
Cleveland College of Chiropractic – Los Angeles	52.1%	31.3%	83.3%
Life University	70.6%	17.3%	87.8%
Life University West	51.7%	29.2%	80.9%
Logan University	45.2%	23.2%	68.4%
National University of Health Sciences	38.7%	25.0%	63.7%
New York Chiropractic College	36.5%	24.0%	60.5%
Northwestern University of Health Sciences	53.3%	24.4%	77.8%
Palmer College of Chiropractic – Iowa	67.8%	16.6%	84.4%
Palmer College of Chiropractic – Florida	51.3%	20.5%	71.8%
Palmer College of Chiropractic – West	43.6%	20.2%	63.8%
Parker University	54.9%	23.5%	78.4%
Southern California University of Health Sciences	45.1%	19.4%	64.6%
Sherman College of Chiropractic	71.2%	19.7%	90.9%
Texas Chiropractic College	57.6%	18.8%	76.5%
University of Bridgeport	25.6%	23.1%	48.7%
University of Western States	28.4%	18.7%	47.1%
***Average total response proportion***	*53.4%*	*20.9%*	*74.3%*

Regarding the prevalence of the use of x-rays for new patients (survey question 7), we found a bimodal pattern in which respondents most commonly reported prescribing x-rays for 0% to 20% of new patients (38.6%). The second most common response indicated was prescribing x-rays for 81% to 100% (20.5%) of new patients. There was widespread variation in the frequency of new patients receiving x-rays.

## Discussion

This study is the first to have evaluated the influences of three clinician-level factors (chiropractic degree program of graduation, years since chiropractic degree completion, and US region of primary practice) on clinical ideologies, beliefs, and practice patterns using data from a large national survey of US chiropractors. These findings are consistent with other international surveys of chiropractors and chiropractic students, supporting the existence of subcultures within the profession regarding differing beliefs and ideologies [6, 25, 31, 32]. Our study is novel because it is the first to suggest that variability in chiropractic intra-professional beliefs and subcultures is explained, in part, by when and where a chiropractor trained and in which region of the US the chiropractor primarily practices.

Several chiropractic degree programs of graduation (Life GA, Life West, Palmer Dav, Parker, and Sherman) consistently had proportions of respondents selecting answers corresponding with the subluxation-based subgroup compared to the total response proportion. Compared to respondents in their first decade of practice, respondents with two, three, and four or more decades of experience had an increasingly greater proportion of responses consistent with the subluxation-based subgroup across all survey items. Those respondents whose primary region of practice was also the location of a chiropractic degree program whose respondents predominantly selected responses consistent with the subluxation-based subgroup did not demonstrate a marked difference in the proportion of responses compared to respondents from other regions of practice. While, primary region of practice might explain some variability, no clear patterns were observed across the 4 regions to suggest any marked differences.

An evaluation of attitudes and characteristics of Canadian chiropractors identified that graduation from specific chiropractic degree programs was associated with a respondent’s membership to differing subgroups [33]. The majority of respondents in this survey identified with the ‘spine and neuromusculoskeletal focused’ subgroup, which was consistent with the subgrouping found in the Canadian evaluation. Substantial variation among respondents from single chiropractic degree programs makes it difficult to suggest one’s chiropractic degree program of graduation is a strong, individual indicator to identify those US chiropractors who are ideal candidates to serve within inter-professional collaborative settings and integrate into team-based healthcare systems. Refinement of a US chiropractor’s attitudes, beliefs, and ideologies to reduce intra-professional variability may need further shaping at—and beyond—the chiropractic degree program of graduation, such as socialization in post-graduate residency within integrated settings.

Two topics, vaccination and use of x-ray to detect a subluxation are known for their divisive nature within the US chiropractic profession when presented to respondents as 5-item Likert scales [2, 6, 10]. In survey design, there is debate regarding the use of a ‘neutral’ or ‘undecided’ choice when asking a question that requires the responder to agree or disagree [34, 35]. For the chiropractic degree program of graduation, we found a large proportion of respondents selected ‘neutral’ for survey items 6 and 7, more than one-fifth (22.6%) and more than one-fourth (28.9%) of respondents, respectively. Because validity and reliability were not extensively evaluated to ensure respondents interpretation and meaning of questions or answer choices, we decided it was necessary to provide two interpretations of the results (Tables 3 and 4). Several meanings may explain a respondent’s choice of ‘neutral’: – satisficing, social desirability bias, or a respondent truly holds no opinion [36].

For ‘vaccination is a positive public health effort’, ‘neutral’ responses ranged from 16.8% to 39.6% of respondents by chiropractic degree program of graduation. Meanwhile ‘neutral’ responses for ‘plain film imaging is helpful in the detection of vertebral subluxations’ ranged from 15.6% to 31.3% by chiropractic degree program of graduation. In addition to discordant beliefs and attitudes with these statements, ambivalence towards these clinical topics should be of concern for the chiropractic profession, as these are possibly key variable beliefs and attitudes that act as barriers to inter-professional integration [10].

The exploration of years since degree completion presented a relatively uniform pattern where recent graduates (respondents 1–10 years since graduation) had a greater proportion of responses that were consistent with being in the spine and neuromusculoskeletal subgroup. As respondents progressed in years since graduation, they were more likely to choose answers consistent with the minority, subluxation-based subgroup, for each topic. There have been drastic changes in chiropractic education over the years that respondents have practiced. The propensity for more experienced practitioners to choose the minority, subluxation-based subgroup could be evidence of that change. Thus, rather than years of experience causing a shift towards subluxation-based practice, it could be that subluxation-based practice was the more common mode of practice taught in past years. Teaching evidence-based practice has become required within chiropractic degree training programs as an accreditation standard and the reduced importance of the subluxation within curricula may be responsible for the shift towards the spine and neuromusculoskeletal subgroup [37–39].

Although data about the quality of care delivered was not captured in this survey, other studies have found a negative correlation between number of years in medical practice and the quality of care that the physician provides [23, 40, 41]. For example, an evaluation of low-value health care services found a negative association between progression of primary care physician age and healthcare quality [42]. These low-value services included stress testing for stable coronary disease, imaging for patients with nonspecific lower back pain in the first 6 weeks, and arthroscopic surgery for knee osteoarthritis.

Our results are an early indication that further work is needed to describe and evaluate the quality of care provided by chiropractors based on years since graduation. It is possible that more recent graduates are exposed to up-to-date basic, medical, and clinical sciences, and are more conversant in evidence-based practice compared to chiropractors in the latter decades of practice. Future work should also consider evaluating the practice behaviors of chiropractors in concordance with clinical practice guidelines and intra-professional characteristics.

For US geographical region of primary practice, there was no consistent trend in variation for the responses to all 7 survey items. At least 50% of respondents from all 4 geographic regions self-identified with the spine and neuromusculoskeletal subgroup, suggesting widespread distribution without clear influence of a regional degree program. If degree program alone were to influence a region, we would have expected the South region to have a far lower proportion neuromusculoskeletal subgroup as both Sherman College of Chiropractic and Life University are within the South region and represent 2 of the 3 lowest respondents degree programs for the spine and neuromusculoskeletal subgroup, while accounting for 14.3% of total survey respondents.

Previous research has found differences in x-ray utilization rates associated with geographical region of practice, but the results of our study did not find this same association [18]. One potential difference that may account for the different findings is that the previous study utilized data from chiropractors who were all enrolled in the same provider network, whereas our study utilized data from a random sample. Another difference that may explain our conflicting results from our survey is that we requested an estimate of x-ray utilization, while the previous survey measured utilization, a trend that may be explained by social-desirability bias. Environmental factors, such as state scope of practice or radiological equipment ownership, were not evaluated and may influence practice clinical ideology, beliefs, and practice patterns more than regional similarities. Specific to our survey items, scope of practice across the US allows for full spine x-ray, suggesting this might have limited influence on our results [43]. Other aspects of clinical ideology, beliefs, or practice patterns could be influenced due to restrictions of scope of practice by several states such as performance of physical exam procedures (e.g., ears, eyes, nose and throat exam, abdominal exam) or imaging techniques (e.g., diagnostic ultrasound) [43]. Further, organizing multiple states to regions for this analysis may have lost resolution for significant differences between states or impact of proximity to a chiropractic degree program but may not be adequately represented due to low respondent rate by state.

## Implications

There are several implications to consider in understanding the wide variation in the attitudes and beliefs of US chiropractors. Per our results, there are a relatively high proportion of attitudes and beliefs among US chiropractors that run contrary to public health recommendations (e.g., vaccination, treatment of patients with cancer with spinal manipulative therapy). These contradictory attitudes and beliefs may potentially cause public confusion and impact safety, preventing the public from seeking much needed preventative services or expend time and money on care that is not supported by the literature. Dissent and ambivalence of the generally accepted positive influence of vaccinations on global health is contrary to sound scientific evidence and public health stance, layering confusion and mixed messaging among healthcare professionals [44, 45]. Chiropractors should provide patients with up-to-date and unbiased information based on sound scientific evidence or recommend the patient speak with their qualified medical physician [46]. Further, guideline discordant clinical care patterns, such as x-ray utilization for 80–100% of new patients regardless of presenting complaint impact the quality of care delivered by the chiropractic profession [47, 48].

This suggests that the variable professional attitudes and beliefs may cause public confusion due to contrasting public health messaging and care patterns. We suggest these features of the chiropractic professions’ attitudes and beliefs detract from—and create barriers to—inter-professional dialogue, integration within team-based environments, and the broader healthcare system.

## Limitations and strengths

We acknowledge several limitations of this study. We selected 3 clinician-level factors for evaluation which does not necessarily limit other clinical-level factors, such as patient volume or knowledge of clinical practice guidelines, from explaining a portion of the variation in attitude and practice behaviors of US chiropractors [25].

The survey instrument was not validated psychometrically prior to deployment, which could have contributed to potential interpretation bias of individual survey items. While face validity was considered, it is possible that undefined and ambiguous terms for certain survey items may have caused confusion and led to differences in interpretation by the respondents. The suboptimal response rate (39.4%) may have impacted the findings and the frequencies of responses should be interpreted with caution, though we made efforts to address institutions with low response rates social-desirability bias may have influenced survey item responses although efforts were made to address this bias by ensuring anonymity with the use of a mail-in survey. By providing respondents with a paper survey for return via mail, we were unable to probe for missing data or incomplete responses and people did not universally follow the directions to ‘select one answer’. In addition, multiple answers were selected by some respondents limiting the interpretation of some answers to survey questions.

Some chiropractic degree programs have closed, and alumni merged with other chiropractic degree programs which may not necessarily represent the beliefs and ideologies of a respondent’s original chiropractic degree program institution. This was a survey of self-reported attitudes and practice patterns only and did not evaluate or verify actual practice patterns which may be incongruent with responses. The clinician-level factors that we evaluated are only three possible considerations and are not necessarily directly reflective of the attitudes and beliefs of the chiropractic degree program of graduation, as many influences on beliefs occur after graduation that include post-graduate education [49].

Despite these limitations, several strengths are present in our study. First, our sampling is consistent with prior demographic reporting of the US chiropractic profession suggesting our results are robust and highly generalizable [50]. Second, we made efforts to promote anonymity of responses to the survey in effort to ensure accurate answers from respondents. Third, to our knowledge this secondary analysis presents the results of the largest random sample survey of US chiropractors, representing all 50 states and the District of Columbia.

## Conclusions

This secondary analysis of a cross-sectional survey (*n* = 3538) of licensed chiropractors in the US revealed unique associations between variations in clinical ideology, beliefs, and practice patterns with chiropractic degree program of graduation, years since chiropractic degree completion, and geographic region of practice. Future work should investigate how these 3 clinician-level factors influence practice variation while not excluding other potential patient-level (e.g. religiosity, socioeconomic status), clinician-level (e.g., weekly practice volume, income, post-graduate education), or environment-level factors (e.g., rurality or state scope of practice).

### Supplementary Information

**Additional file 1: Appendix 1A.** Multinomial logistic regression models 95% confidence interval output for the relative risk ratio given the other predictors are in the model: chiropractic degree program of graduation. **Appendix 1B.** Table Multinomial logistic regression models 95% confidence interval output for the relative risk ratio given the other predictors are in the model: years since of chiropractic degree completion. **Appendix 1C.** Multinomial logistic regression models 95% confidence interval output for the relative risk ratio given the other predictors are in the model: primary practice location (US Census region).

**Additional file 2: Appendix 2A.** Association between years since completion of chiropractic degree and ideologies, beliefs, and practice patterns. **Appendix 2B.** Association between region of primary practice location and ideologies, beliefs, and practice patterns.

## Data Availability

The dataset used and analyzed during the current study are available from the corresponding author upon reasonable request.

## Abbreviation

US: United States

## References

[1] Meeker WC, Haldeman S (2002). Chiropractic: a profession at the crossroads of mainstream and alternative medicine. Ann Intern Med.

[2] Gliedt JA, Perle SM, Puhl AA, Daehler S, Schneider MJ, Stevans J (2021). Evaluation of United States chiropractic professional subgroups: a survey of randomly sampled chiropractors. BMC Health Serv Res.

[3] The Future of Chiropractic Revisited: 2005 to 2015. Alexandria: Institute for Alternative Futures; 2005. Available from: http://www.altfutures.com.

[4] Mccallin A (2001). Interdisciplinary practice - a matter of teamwork: an integrated literature review. J Clin Nurs.

[5] Liberati EG, Gorli M, Scaratti G (2016). Invisible walls within multidisciplinary teams: disciplinary boundaries and their effects on integrated care. Soc Sci Med.

[6] McGregor M, Puhl AA, Reinhart C, Injeyan HS, Soave D (2014). Differentiating intraprofessional attitudes toward paradigms in health care delivery among chiropractic factions: results from a randomly sampled survey. BMC Complement Altern Med.

[7] Mitchell RJ, Parker V, Giles M (2011). When do interprofessional teams succeed? Investigating the moderating roles of team and professional identity in interprofessional effectiveness. Hum Relat.

[8] Morgan PI, Ogbonna E (2008). Subcultural dynamics in transformation: a multi-perspective study of healthcare professionals. Hum Relat.

[9] Bajwa NM, Bochatay N, Muller-Juge V, Cullati S, Blondon KS, JunodPerron N (2020). Intra versus interprofessional conflicts: implications for conflict management training. J Interprof Care.

[10] Leboeuf-Yde C, Innes SI, Young KJ, Kawchuk GN, Hartvigsen J (2019). Chiropractic, one big unhappy family: better together or apart?. Chiropr Man Ther.

[11] Elton D, Kosloff T. Using big data to advance value-based spine care. Spineline (North American Spine Society); 2015. p. 17–22.

[12] Fritz JM, Kim J, Dorius J (2016). Importance of the type of provider seen to begin health care for a new episode low back pain: associations with future utilization and costs: entry provider for low back pain care. J Eval Clin Pract.

[13] Kosloff TM, Elton D, Shulman SA, Clarke JL, Skoufalos A, Solis A (2013). Conservative spine care: opportunities to improve the quality and value of care. Popul Health Manag.

[14] Busse JW, Jacobs C, Ngo T, Rodine R, Torrance D, Jim J (2009). Attitudes toward chiropractic: a survey of North American orthopedic surgeons. Spine.

[15] Lisi AJ, Khorsan R, Smith MM, Mittman BS (2014). Variations in the Implementation and Characteristics of Chiropractic Services in VA. Med Care.

[16] Schneider M, Murphy D, Hartvigsen J (2016). Spine care as a framework for the chiropractic identity. J Chiropr Humanit.

[17] Weeks WB, Goertz CM, Meeker WC, Marchiori DM (2016). Characteristics of US adults who have positive and negative perceptions of doctors of chiropractic and chiropractic care. J Manipulative Physiol Ther.

[18] Bussières AE, Sales AE, Ramsay T, Hilles S, Grimshaw JM (2013). Practice patterns in spine radiograph utilization among doctors of chiropractic enrolled in a provider network offering complementary care in the United States. J Manipulative Physiol Ther.

[19] O’Neill L, Kuder J (2005). Explaining variation in physician practice patterns and their propensities to recommend services. Med Care Res Rev.

[20] Reschovsky JD, Rich EC, Lake TK (2015). Factors contributing to variations in physicians’ use of evidence at the point of care: a conceptual model. J Gen Intern Med.

[21] Burns LR, Wholey DR (1991). The effects of patient, hospital, and physician characteristics on length of stay and mortality. Med Care.

[22] Van Parys J (2016). Variation in physician practice styles within and across emergency departments. PLoS One.

[23] Choudhry NK, Fletcher RH, Soumerai SB (2005). Systematic review: the relationship between clinical experience and quality of health care. Ann Intern Med.

[24] Keating NL, Huskamp HA, Kouri E, Schrag D, Hornbrook MC, Haggstrom DA (2018). Factors contributing to geographic variation in end-of-life expenditures for cancer patients. Health Aff (Millwood).

[25] Gíslason HF, Salminen JK, Sandhaugen L, Storbråten AS, Versloot R, Roug I (2019). The shape of chiropractic in Europe: a cross sectional survey of chiropractor’s beliefs and practice. Chiropr Man Ther.

[26] Swain MS, Gliedt JA, de Luca K, Newell D, Holmes M (2021). Chiropractic students’ cognitive dissonance to statements about professional identity, role, setting and future: international perspectives from a secondary analysis of pooled data. Chiropr Man Ther.

[27] Blanchette MA, Engmark N, Sørensen MM, Mior S, Stochkendahl MJ (2021). Association between characteristics of danish chiropractors and number of referred patients from general practitioners: a cross-sectional study. J Manipulative Physiol Ther.

[28] Stochkendahl MJ, Rezai M, Torres P, Sutton D, Tuchin P, Brown R (2019). The chiropractic workforce: a global review. Chiropr Man Ther.

[29] Sharma A, Minh Duc NT, Luu Lam Thang T, Nam NH, Ng SJ, Abbas KS (2021). A Consensus-Based Checklist for Reporting of Survey Studies (CROSS). J Gen Intern Med.

[30] United States Census Bureau. Geographic Levels. Available from: https://www.census.gov/programs-surveys/economic-census/guidance-geographies/levels.html; [cited 15 Jan 2022].

[31] Gliedt JA, Hawk C, Anderson M, Ahmad K, Bunn D, Cambron J (2015). Chiropractic identity, role and future: a survey of North American chiropractic students. Chiropr Man Ther.

[32] de Luca KE, Gliedt JA, Fernandez M, Kawchuk G, Swain MS (2018). The identity, role, setting, and future of chiropractic practice: a survey of Australian and New Zealand chiropractic students. J Chiropr Educ.

[33] Puhl AA, Reinhart CJ, Doan JB, McGregor M, Injeyan HS (2014). Relationship between chiropractic teaching institutions and practice characteristics among Canadian doctors of chiropractic: a random sample survey. J Manipulative Physiol Ther.

[34] Kalton G, Roberts J, Holt D (1980). The effects of offering a middle response option with opinion questions. Statistician.

[35] Presser S, Schuman H (1980). The measurement of a middle position in attitude surveys. Public Opin Q.

[36] Krosnick JA (1991). Response strategies for coping with the cognitive demands of attitude measures in surveys. Appl Cogn Psychol.

[37] Innes SI, Leboeuf-Yde C, Walker BF (2016). How comprehensively is evidence-based practice represented in Councils on Chiropractic Education (CCE) educational standards: a systematic audit. Chiropr Man Ther.

[38] Mirtz TA, Perle SM (2011). The prevalence of the term subluxation in North American English-Language Doctor of chiropractic programs. Chiropr Man Ther.

[39] Funk MF, Frisina-Deyo AJ, Mirtz TA, Perle SM (2018). The prevalence of the term subluxation in chiropractic degree program curricula throughout the world. Chiropr Man Ther.

[40] Ajmi SC, Aase K (2021). Physicians’ clinical experience and its association with healthcare quality: a systematised review. BMJ Open Qual.

[41] Blasier RB (2009). The Problem of the aging surgeon: when surgeon age becomes a surgical risk factor. Clin Orthop.

[42] Schwartz AL, Jena AB, Zaslavsky AM, McWilliams JM (2019). Analysis of physician variation in provision of low-value services. JAMA Intern Med.

[43] Chang M (2014). The chiropractic scope of practice in the United States: a cross-sectional survey. J Manipulative Physiol Ther.

[44] Pulendran B, Ahmed R (2011). Immunological mechanisms of vaccination. Nat Immunol.

[45] Filice E, Dubé E, Graham JE, MacDonald NE, Bettinger JA, Greyson D (2020). Vaccination discourses among chiropractors, naturopaths and homeopaths: a qualitative content analysis of academic literature and Canadian organizational webpages. PLOS One.

[46] Khorsan R, Smith M, Hawk C, Haas M (2009). A public health immunization resource web site for chiropractors: discussion of current issues and future challenges for evidence-based initiatives for the chiropractic profession. J Manipulative Physiol Ther.

[47] Bussières AE, Taylor JAM, Peterson C (2008). Diagnostic imaging practice guidelines for musculoskeletal complaints in adults—an evidence-based approach—part 3: spinal disorders. J Manipulative Physiol Ther.

[48] Chou R (2011). Diagnostic imaging for low back pain: advice for high-value health care from the American college of physicians. Ann Intern Med.

[49] Clayton GM, Broome ME, Ellis LA (1989). Relationship between preceptorship experience and roles socialization of graduate nurses. J Nurs Educ.

[50] Himelfarb I, Hyland J, Ouzts N, Russell M, Sterling T, Johnson C, et al. Practice Analysis of Chiropractic 2020 - A project report, survey analysis, and summary of the practice if chiropractic within the United States. Greeley: National Board of Chiropractic Examiners. Available from: https://mynbce.org/wp-content/uploads/2020/02/Executive-Summary-Practice-Analysis-of-Chiropractic-2020.pdf; [cited 5 Jun 2020].

