# Correction: Associations between demographics and clinical ideology, beliefs, and practice patterns: a secondary analysis of a survey of randomly sampled United States chiropractors

**DOI:** 10.1186/s12906-023-04265-5

**Published:** 2023-11-25

**Authors:** Zachary A. Cupler, Jordan A. Gliedt, Stephen M. Perle, Aaron A. Puhl, Michael J. Schneider

**Affiliations:** 1 Butler VA Health Care System, Butler, PA USA; 2 Institute for Clinical Research Education, University of Pittsburgh, Pittsburgh, PA USA; 3 Department of Neurosurgery, Medical College of Wisconsin, Milwaukee, WI USA; 4 Big Data Interrogation Group, AECC University College, Bournemouth, Dorset UK; 5 Discipline of Chiropractic, College of Science, Health, Engineering and Education, Murdoch University, Murdoch, WA Australia; 6 Private Practice, Able Body Health Clinic, Lethbridge, AB Canada; 7 Department of Physical Therapy, University of Pittsburgh, Pittsburgh, PA USA; 8 Clinical and Translational Science Institute, University of Pittsburgh, Pittsburgh, PA USA

**Correction: BMC Complement Med Ther 23, 404 (2023)**

10.1186/s12906-023-04225-z

Following publication of the original article [1], the authors identified an error in Fig. 2 caption. The legend/key are missing. The correct Figure 2 caption is given below.

## Fig. 2

Stacked bar graphs representing association with chiropractic degree program of graduation and ideologies, beliefs, and practice patterns

Each bar graph represents the sum of all response by the labeled subgroup on the x-axis and the color, matched to each graphs respective key, is the proportion of respondents within the subgroup who selected each answer and only identified a single correct answer. Respondents who answered for more than once choice were not represented in the bar graphs.

Palmer: Palmer College of Chiropractic Main Campus, Davenport, IA; CCC-KC: Cleveland University Overland Park, KS; CCC-LA: Cleveland Chiropractic College Los Angles; Life: Life University, GA; Life West: Life Chiropractic College West Hayward, CA; Logan: Logan University, Chesterfield, MO; National: National University of Health Sciences, Lombard IL and Seminole, FL; Northwestern: Northwestern Health Sciences University, Bloomington, MN; NYCC: Northeast College of Health Sciences (formerly New York Chiropractic College), Seneca Falls, NY; Palmer-FL; Palmer College Of Chiropractic Florida Campus, Port Orange, FL; Palmer-West: Palmer College of Chiropractic West Campus, San Jose, CA; Parker: Parker University, Dallas, TX; SCUHS: Southern California University of Health Sciences, Whittier, CA; Sherman: Sherman College of Chiropractic, Spartanburg, SC; TCC: Texas Chiropractic College, Pasadena, TX; Bridgeport: University of Bridgeport, Bridgeport, CT; Western States: University of Western States, Portland, OR

Survey Question #1 labels: DDx: Differential Diagnosis only; DDx > SA: Focus on differential diagnosis, sometimes includes spinal analysis; DDx + SA: Equal focus on spinal analysis to detect subluxation and differential diagnosis; SA > DDx: Focus on Spinal analysis, sometimes includes differential diagnosis; SA: Spinal analysis to detect subluxation only

Survey Question #2 labels: nMSK: Neuromusculoskeletal Conditions; MSKgen: General and Biomechanical Conditions; MSKsub: Vertebral Subluxation as a Musculoskeletal Condition; Somatovisc: Biomechanical and Organic/Visceral Conditions; Broad: Broad Spectrum of Health Concerns Including Lifestyle and Wellness Issues; VS: Vertebral Subluxation as an Encumbrance to Health

Survey Question #3 labels: NMSK: spine and neuromusculoskeletal focused subgroup; Primary Care: General primary care focused subgroup; Subluxation: Subluxation detection and removal subgroup

Survey Question #4 labels: None: No Role; QoL: Improving Pain/Quality of Life; ImmuneFx: Improving Nervous System/Immune System Function; Innate: Removing Interference to Innate Intelligence

Survey Question #5 labels: SA + A: Strongly Agree and Agree responses; SD + D: Disagree and Strongly Disagree responses

Survey Question #6 labels: SD + D: Strongly Disagree and Disagree responses; A + SA: Agree and Strongly Agree responses

The original article has been corrected.
