# Supplementary material for: Associations between demographics and clinical ideology, beliefs, and practice patterns: a secondary analysis of a survey of randomly sampled United States chiropractors
Source: BMC Complement Med Ther. 2023 Nov 9;23:404. doi: 10.1186/s12906-023-04225-z (PMC10634061; doi:10.1186/s12906-023-04225-z)
Supplement: Supplementary file 1 — Additional file 1: Appendix 1A. Multinomial logistic regression models 95% confidence interval output for the relative risk ratio given the other predictors are in the model: chiropractic degree program of graduation. Appendix 1B. Table Multinomial logistic regression models 95% confidence interval output for the relative risk ratio given the other predictors are in the model: years since of chiropractic degree completion. Appendix 1C. Multinomial logistic regression models 95% confidence interval output for the relative risk ratio given the other predictors are in the model: primary practice location (US Census region). [file 12906_2023_4225_MOESM1_ESM.zip › Appendix_1A_Colleges_table_v1__7.31.2023.docx]

Appendix 1A. Multinomial logistic regression models 95% confidence interval output for the relative risk ratio given the other predictors are in the model: chiropractic degree program of graduation

|  | Palmer | Cleveland College of Chiropractic - Kansas City | | | Cleveland College of Chiropractic – Los Angeles | | | Life University | | | Life University West | | | Logan University | | | National University of Health Sciences | | |
| --- | --- | --- | --- | --- | --- | --- | --- | --- | --- | --- | --- | --- | --- | --- | --- | --- | --- | --- | --- |
| Q1: Scope of Examination^1^ | BASE OUTCOME | *p* | 95% CI | | *p* | 95% CI | | *p* | 95% CI | | *p* | 95% CI | | *p* | 95% CI | | *p* | 95% CI | |
| SA |  | -- | -- | -- | -- | -- | -- | -- | -- | -- | -- | -- | -- | -- | -- | -- | -- | -- | -- |
| SA>DDx |  | .20 | .66 | 7.06 | .99 | 0 | . | .73 | .41 | 1.87 | .73 | .36 | 4.25 | .57 | .51 | 3.36 | .02 | 1.32 | 49.65 |
| SA+DDx |  | .05 | .99 | 4.17 | .07 | .87 | 47.96 | .03 | .47 | .95 | .38 | .40 | 1.42 | .04 | 1.02 | 2.75 | .00 | 3.69 | 62.38 |
| DDx>SA |  | .29 | .49 | 10.98 | .00 | 4.23 | 2500.93 | .08 | .05 | 1.18 | .99 | 0 | . | .09 | .87 | 8.19 | .00 | 5.37 | 213.35 |
| DDX |  | .36 | .56 | 4.80 | .18 | .49 | 63.83 | .02 | .21 | .88 | .14 | .07 | 1.46 | .00 | 1.73 | 6.65 | .00 | 12.25 | 236.29 |
| State |  | .00 | .98 | .99 | <.001 | .93 | .97 | .00 | .98 | .99 | .00 | .94 | .97 | .10 | .99 | 1.00 | .02 | .98 | 1.00 |
| Gender |  | .81 | .62 | 1.46 | .02 | 1.07 | 2.30 | .69 | .70 | 1.27 | .25 | .84 | 1.98 | .71 | .68 | 1.30 | .25 | .87 | 1.70 |
| Years in Practice |  | <.001 | .62 | .85 | .24 | .90 | 1.50 | .00 | .71 | .88 | .00 | .57 | .84 | .00 | .70 | .88 | .00 | 1.10 | 1.42 |
| Survey Type |  | .61 | .67 | 1.99 | .11 | .03 | 1.43 | .89 | .69 | 1.52 | .85 | .53 | 2.13 | .57 | .75 | 1.70 | .35 | .48 | 1.30 |
| constant |  | .01 | .15 | .76 | .00 | .00 | .32 | .00 | 1.26 | 3.14 | .65 | .56 | 2.54 | .03 | .29 | .94 | .00 | .00 | .08 |
| Q2: Conditions Treated^2^ | BASE OUTCOME | *p* | 95% CI | | *p* | 95% CI | | *p* | 95% CI | | *p* | 95% CI | | *p* | 95% CI | | *p* | 95% CI | |
| VS |  | -- | -- | -- | -- | -- | -- | -- | -- | -- | -- | -- | -- | -- | -- | -- | -- | -- | -- |
| Broad |  | .19 | .83 | 2.50 | .24 | .69 | 4.42 | .05 | .49 | 1.00 | .58 | .42 | 1.63 | <.001 | 1.75 | 4.28 | <.001 | 3.94 | 16.71 |
| Somatovisc |  | .20 | .73 | 4.31 | .38 | .45 | 7.88 | .50 | .43 | 1.51 | .74 | .38 | 3.86 | .01 | 1.36 | 5.48 | <.001 | 4.03 | 23.53 |
| MSKsub |  | .46 | .31 | 1.69 | .89 | .23 | 3.66 | .04 | .37 | .98 | .28 | .71 | 3.29 | .26 | .77 | 2.63 | .00 | 1.59 | 9.15 |
| MSKgen |  | .02 | 1.12 | 4.02 | .98 | .24 | 3.96 | .05 | .37 | 1.01 | .82 | .47 | 2.58 | <.001 | 1.57 | 4.65 | <.001 | 2.22 | 11.87 |
| nMSK |  | .42 | .72 | 2.20 | .13 | .80 | 5.20 | .03 | .47 | .97 | .86 | .55 | 2.03 | <.001 | 1.67 | 4.10 | <.001 | 5.33 | 22.50 |
| State |  | .002 | .98 | .99 | <.001 | .93 | .97 | <.001 | .98 | .99 | <.001 | .94 | .97 | .09 | .99 | 1.00 | .03 | .98 | 1.00 |
| Gender |  | .78 | .60 | 1.46 | .02 | 1.07 | 2.37 | .91 | .72 | 1.33 | .17 | .88 | 2.12 | .58 | .65 | 1.27 | .31 | .85 | 1.68 |
| Years in Practice |  | <.001 | .61 | .84 | .21 | .91 | 1.53 | <.001 | .71 | .89 | <.001 | .56 | .84 | <.001 | .68 | .86 | .01 | 1.06 | 1.37 |
| Survey Type |  | .40 | .77 | 1.94 | .12 | .12 | 1.27 | .91 | .70 | 1.38 | .78 | .61 | 1.94 | .42 | .81 | 1.65 | .48 | .56 | 1.32 |
| constant |  | .02 | .27 | .91 | <.001 | .05 | .42 | .00 | 1.24 | 2.79 | .69 | .44 | 1.73 | .00 | .27 | .74 | <.001 | .02 | .11 |

**Palmer**: Palmer College of Chiropractic – Davenport, Iowa

^1^ Survey Question 1 labels: **SA:** Spinal analysis to detect subluxation only; **SA>DDx:** Focus on Spinal analysis, sometimes includes differential diagnosis; **SA+DDx:** Equal focus on spinal analysis to detect subluxation and differential diagnosis; **DDx>SA:** Focus on differential diagnosis, sometimes includes spinal analysis; **DDx:** Differential Diagnosis only

^2^ Survey Question 2 labels: **VS:** Vertebral Subluxation as an Encumbrance to Health; **Broad:** Broad Spectrum of Health Concerns Including Lifestyle and Wellness Issues; **Somatovisc:** Biomechanical and Organic/Visceral Conditions; **MSKsub:** Vertebral Subluxation as a Musculoskeletal Condition; **MSKgen:** General and Biomechanical Conditions; **nMSK:** Neuromusculoskeletal Conditions

|  | Palmer | Northwestern University of Health Sciences | | | New York Chiropractic College | | | Palmer College of Chiropractic – Florida | | | Palmer College of Chiropractic – West | | | Parker University | | | Southern California University of Health Sciences | | |
| --- | --- | --- | --- | --- | --- | --- | --- | --- | --- | --- | --- | --- | --- | --- | --- | --- | --- | --- | --- |
| Q1: Scope of Examination^1^ | BASE OUTCOME | *p* | 95% CI | | *p* | 95% CI | | *p* | 95% CI | | *p* | 95% CI | | *p* | 95% CI | | *p* | 95% CI | |
| SA |  | -- | -- | -- | -- | -- | -- | -- | -- | -- | -- | -- | -- | -- | -- | -- | -- | -- | -- |
| SA>DDx |  | .49 | .49 | 4.39 | .06 | .96 | 8.04 | .29 | .30 | 52.28 | .99 | 0 | . | .69 | .18 | 3.08 | .86 | .15 | 4.70 |
| SA+DDx |  | .01 | 1.18 | 3.81 | <.001 | 1.68 | 6.15 | .09 | .76 | 42.88 | .02 | 1.33 | 14.12 | .31 | .76 | 2.41 | .01 | 1.32 | 6.03 |
| DDx>SA |  | .47 | .39 | 7.63 | .03 | 1.12 | 15.71 | .99 | 0 | . | .99 | 0 | . | .91 | .20 | 6.11 | .02 | 1.31 | 26.85 |
| DDX |  | .23 | .71 | 4.19 | <.001 | 2.92 | 14.58 | .39 | .25 | 34.61 | .01 | 1.47 | 23.99 | .59 | .52 | 3.16 | .001 | 1.92 | 13.06 |
| State |  | .71 | .99 | 1.01 | .01 | 1.00 | 1.02 | .31 | .97 | 1.01 | <.001 | .95 | .97 | <.001 | 1.02 | 1.04 | <.001 | .94 | .96 |
| Gender |  | .06 | .99 | 1.82 | .30 | .86 | 1.63 | .56 | .63 | 2.34 | .27 | .83 | 1.94 | .89 | .69 | 1.53 | .23 | .87 | 1.76 |
| Years in Practice |  | <.001 | .54 | .72 | .68 | .86 | 1.10 | <.001 | .08 | .24 | <.001 | .57 | .84 | <.001 | .48 | .66 | .61 | .83 | 1.11 |
| Survey Type |  | .85 | .66 | 1.65 | .99 | .65 | 1.56 | .50 | .58 | 3.11 | .75 | .45 | 1.77 | .47 | .47 | 1.42 | .52 | .47 | 1.47 |
| constant |  | .00 | .16 | .64 | <.001 | .04 | .19 | .03 | .01 | .80 | .02 | .06 | .73 | <.001 | .08 | .37 | .06 | .19 | 1.02 |
| Q2: Conditions Treated^2^ | BASE OUTCOME | *p* | 95% CI | | *p* | 95% CI | | *p* | 95% CI | | *p* | 95% CI | | *p* | 95% CI | | *p* | 95% CI | |
| VS |  | -- | -- | -- | -- | -- | -- | -- | -- | -- | -- | -- | -- | -- | -- | -- | -- | -- | -- |
| Broad |  | <.001 | 1.66 | 4.31 | <.001 | 1.49 | 3.97 | .37 | .58 | 4.40 | .14 | .83 | 3.84 | .08 | .94 | 2.71 | .00 | 1.64 | 5.92 |
| Somatovisc |  | .00 | 1.65 | 6.99 | .24 | .71 | 3.87 | .98 | 0 | . | .11 | .82 | 7.96 | .59 | .49 | 3.46 | .16 | .75 | 6.03 |
| MSKsub |  | .14 | .85 | 3.06 | .99 | .48 | 2.09 | .34 | .04 | 2.98 | .91 | .35 | 3.21 | .30 | .73 | 2.81 | .08 | .93 | 4.78 |
| MSKgen |  | .11 | .88 | 3.17 | <.001 | 1.81 | 5.65 | .54 | .41 | 5.43 | .09 | .89 | 5.50 | .19 | .80 | 3.04 | .07 | .95 | 4.91 |
| nMSK |  | .09 | .93 | 2.58 | <.001 | 2.54 | 6.53 | .24 | .69 | 4.57 | .00 | 1.59 | 6.62 | .29 | .78 | 2.26 | <.001 | 2.46 | 8.59 |
| State |  | .60 | .99 | 1.01 | .01 | 1.00 | 1.02 | .38 | .97 | 1.01 | <.001 | .95 | .97 | <.001 | 1.02 | 1.03 | <.001 | .94 | .96 |
| Gender |  | .12 | .94 | 1.77 | .17 | .91 | 1.73 | .38 | .70 | 2.57 | .11 | .92 | 2.10 | .92 | .68 | 1.53 | .24 | .86 | 1.80 |
| Years in Practice |  | <.001 | .53 | .70 | .87 | .88 | 1.12 | <.001 | .08 | .27 | <.001 | .57 | .85 | <.001 | .47 | .66 | .79 | .84 | 1.14 |
| Survey Type |  | .92 | .68 | 1.53 | .90 | .70 | 1.51 | .60 | .57 | 2.67 | .18 | .32 | 1.24 | .2 | .44 | 1.19 | .47 | .50 | 1.37 |
| constant |  | .00 | .21 | .66 | <.001 | .06 | .20 | .03 | .11 | .91 | .04 | .20 | .95 | <.001 | .10 | .35 | .01 | .19 | .77 |

**Palmer**: Palmer College of Chiropractic – Davenport, Iowa

^1^ Survey Question 1 labels: **SA:** Spinal analysis to detect subluxation only; **SA>DDx:** Focus on Spinal analysis, sometimes includes differential diagnosis; **SA+DDx:** Equal focus on spinal analysis to detect subluxation and differential diagnosis; **DDx>SA:** Focus on differential diagnosis, sometimes includes spinal analysis; **DDx:** Differential Diagnosis only

^2^ Survey Question 2 labels: **VS:** Vertebral Subluxation as an Encumbrance to Health; **Broad:** Broad Spectrum of Health Concerns Including Lifestyle and Wellness Issues; **Somatovisc:** Biomechanical and Organic/Visceral Conditions; **MSKsub:** Vertebral Subluxation as a Musculoskeletal Condition; **MSKgen:** General and Biomechanical Conditions; **nMSK:** Neuromusculoskeletal Conditions

|  | Palmer | Sherman College of Chiropractic | | | Texas Chiropractic College | | | University of Bridgeport | | | University of Western States | | |
| --- | --- | --- | --- | --- | --- | --- | --- | --- | --- | --- | --- | --- | --- |
| Q1: Scope of Examination^1^ | BASE OUTCOME | *p* | 95% CI | | *p* | 95% CI | | *p* | 95% CI | | *p* | 95% CI | |
| SA |  | -- | -- | -- | -- | -- | -- | -- | -- | -- | -- | -- | -- |
| SA>DDx |  | .61 | .20 | 2.60 | 1.00 | .09 | 10.76 | .72 | .20 | 10.69 | .11 | .71 | 25.91 |
| SA+DDx |  | <.001 | .12 | .38 | .04 | 1.04 | 11.14 | .39 | .50 | 5.98 | .001 | .25 | 24.52 |
| DDx>SA |  | .76 | .13 | 4.44 | .12 | .68 | 37.41 | .54 | .18 | 26.91 | .001 | 3.36 | 119.68 |
| DDX |  | .99 | 0 | . | .001 | 2.54 | 36.79 | .19 | .60 | 14.50 | ,.001 | 5.88 | 80.84 |
| State |  | .04 | 1.00 | 1.03 | <.001 | 1.02 | 1.04 | .01 | .96 | .99 | .002 | 1.01 | 1.03 |
| Gender |  | .06 | .99 | 2.38 | .96 | .57 | 1.80 | .99 | .47 | 2.09 | .12 | .91 | 1.97 |
| Years in Practice |  | .59 | .86 | 1.31 | .53 | .88 | 1.29 | <.001 | .30 | .60 | <.001 | .61 | .84 |
| Survey Type |  | .94 | .42 | 2.24 | .52 | .64 | 2.43 | .22 | .74 | 3.84 | .43 | .44 | 1.42 |
| constant |  | <.001 | .06 | .36 | <.001 | .002 | .03 | .01 | .04 | .60 | <.001 | .004 | .06 |
| Q2: Conditions Treated^2^ | BASE OUTCOME | *p* | 95% CI | | *p* | 95% CI | | *p* | 95% CI | | *p* | 95% CI | |
| VS |  | -- | -- | -- | -- | -- | -- | -- | -- | -- | -- | -- | -- |
| Broad |  | .05 | .26 | 1.01 | .00 | 1.75 | 12.71 | .03 | 1.15 | 11.78 | <.001 | 3.10 | 16.88 |
| Somatovisc |  | .11 | .03 | 1.48 | .01 | 1.45 | 19.70 | .51 | .22 | 20.37 | .02 | 1.23 | 15.19 |
| MSKsub |  | .90 | .50 | 2.19 | .05 | 1.01 | 10.84 | .68 | .25 | 8.34 | .17 | .70 | 7.12 |
| MSKgen |  | .02 | .01 | .67 | <.001 | 3.22 | 25.23 | .09 | .84 | 13.13 | <.001 | 4.45 | 27.31 |
| nMSK |  | .02 | .18 | .84 | .00 | 1.69 | 12.49 | .06 | .98 | 10.26 | <.001 | 3.94 | 21.20 |
| State |  | .03 | 1.00 | 1.03 | <.001 | 1.02 | 1.04 | .01 | .96 | .99 | .00 | 1.01 | 1.03 |
| Gender |  | .03 | 1.05 | 2.44 | .78 | .51 | 1.66 | .95 | .49 | 2.15 | .14 | .91 | 1.98 |
| Years in Practice |  | .73 | .84 | 1.29 | .82 | .84 | 1.25 | <.001 | .31 | .60 | <.001 | .60 | .83 |
| Survey Type |  | .42 | .69 | 2.45 | .65 | .63 | 2.11 | .12 | .86 | 3.82 | .61 | .52 | 1.46 |
| constant |  | <.001 | .03 | .18 | <.001 | .00 | .03 | <.001 | .03 | .34 | <.001 | .01 | .05 |

**Palmer**: Palmer College of Chiropractic – Davenport, Iowa

^1^ Survey Question 1 labels: **SA:** Spinal analysis to detect subluxation only; **SA>DDx:** Focus on Spinal analysis, sometimes includes differential diagnosis; **SA+DDx:** Equal focus on spinal analysis to detect subluxation and differential diagnosis; **DDx>SA:** Focus on differential diagnosis, sometimes includes spinal analysis; **DDx:** Differential Diagnosis only

^2^ Survey Question 2 labels: **VS:** Vertebral Subluxation as an Encumbrance to Health; **Broad:** Broad Spectrum of Health Concerns Including Lifestyle and Wellness Issues; **Somatovisc:** Biomechanical and Organic/Visceral Conditions; **MSKsub:** Vertebral Subluxation as a Musculoskeletal Condition; **MSKgen:** General and Biomechanical Conditions; **nMSK:** Neuromusculoskeletal Conditions

|  | Palmer | Cleveland College of Chiropractic – Kansas City | | | Cleveland College of Chiropractic – Los Angeles | | | Life University | | | Life University West | | | Logan University | | | National University of Health Sciences | | | |
| --- | --- | --- | --- | --- | --- | --- | --- | --- | --- | --- | --- | --- | --- | --- | --- | --- | --- | --- | --- | --- |
| Q3: Role in Healthcare System^3^ | BASE OUTCOME | *p* | 95% CI | | *p* | 95% CI | | *p* | 95% CI | | *p* | 95% CI | | *p* | 95% CI | | *p* | 95% CI | | |
| Subluxation |  | -- | -- | -- | -- | -- | -- | -- | -- | -- | -- | -- | -- | -- | -- | -- | -- | -- | -- | |
| Primary Care |  | <.001 | 1.79 | 6.08 | .01 | 1.59 | 12.84 | .48 | .61 | 1.27 | .44 | .36 | 1.56 | <.001 | 1.91 | 4.66 | <.001 | 4.81 | 17.05 | |
| Spine/nMSK |  | <.001 | 1.61 | 4.65 | .02 | 1.26 | 8.87 | .03 | .55 | .97 | .80 | .64 | 1.78 | <.001 | 1.89 | 4.00 | <.001 | 4.04 | 13.23 | |
| State |  | .00 | .97 | .99 | <.001 | .93 | .97 | <.001 | .98 | .99 | <.001 | .94 | .96 | .12 | .99 | 1.00 | .03 | .98 | 1.00 | |
| Gender |  | .80 | .61 | 1.47 | .03 | 1.05 | 2.29 | .96 | .73 | 1.34 | .29 | .81 | 2.03 | .57 | .65 | 1.26 | .38 | .83 | 1.63 | |
| Years in Practice |  | <.001 | .61 | .84 | .36 | .87 | 1.45 | <.001 | .72 | .90 | <.001 | .57 | .85 | <.001 | .69 | .87 | .01 | 1.04 | 1.34 | |
| Survey Type |  | .34 | .79 | 2.00 | .11 | .11 | 1.24 | .98 | .72 | 1.40 | .76 | .61 | 1.95 | .45 | .80 | 1.63 | .34 | .53 | 1.25 | |
| constant |  | <.001 | .15 | .54 | <.001 | .03 | .27 | .02 | 1.07 | 2.33 | .91 | .51 | 1.83 | <.001 | .25 | .66 | <.001 | .03 | .12 | |
| Q4: Role of SMT in Cancer Treatment^4^ | BASE OUTCOME | *p* | 95% CI | | *p* | 95% CI | | *p* | 95% CI | | *p* | 95% CI | | *p* | 95% CI | | *p* | 95% CI | | |
| Innate |  | -- | -- | -- | -- | -- | -- | -- | -- | -- | -- | -- | -- | -- | -- | -- | -- | -- | | -- |
| ImmuneFx |  | .52 | .68 | 2.19 | .07 | .88 | 16.76 | .21 | .88 | 1.83 | .99 | .52 | 1.88 | .00 | 1.50 | 4.54 | .14 | .86 | | 2.98 |
| QoL |  | .19 | .82 | 2.69 | .08 | .85 | 17.04 | .53 | .59 | 1.31 | .46 | .39 | 1.53 | <.001 | 2.42 | 7.29 | <.001 | 2.93 | | 9.64 |
| None |  | .22 | .66 | 6.41 | .00 | 3.55 | 107.74 | .80 | .46 | 2.73 | .82 | .17 | 4.06 | .01 | 1.45 | 10.17 | <.001 | 2.65 | | 17.13 |
| State |  | .00 | .97 | .99 | <.001 | .93 | .97 | <.001 | .98 | .99 | <.001 | .94 | .97 | .07 | .99 | 1.00 | .02 | .98 | | 1.00 |
| Gender |  | .95 | .64 | 1.52 | .01 | 1.17 | 2.50 | .95 | .73 | 1.34 | .25 | .83 | 2.04 | .65 | .66 | 1.29 | .24 | .87 | | 1.74 |
| Years in Practice |  | <.001 | .61 | .85 | .26 | .90 | 1.50 | <.001 | .71 | .88 | <.001 | .56 | .84 | <.001 | .69 | .88 | .00 | 1.10 | | 1.42 |
| Survey Type |  | .37 | .78 | 1.96 | .11 | .11 | 1.24 | .85 | .74 | 1.45 | .66 | .64 | 2.03 | .52 | .79 | 1.60 | .36 | .53 | | 1.26 |
| constant |  | .04 | .25 | .98 | <.001 | .01 | .27 | .35 | .79 | 1.98 | .97 | .47 | 2.16 | <.001 | .17 | .58 | <.001 | .05 | | .20 |

**Palmer**: Palmer College of Chiropractic – Davenport, Iowa

^3^Survey Question 3 labels**: nMSK:** neuromusculoskeletal conditions

^4^Survey Question 4 labels: **Innate:** Removing Interference to Innate Intelligence; **ImmuneFx:** Improving Nervous System/Immune System Function; **QoL:** Improving Pain/Quality of Life; **None:** No Role

|  | Palmer | Northwestern University of Health Sciences | | | New York Chiropractic College | | | Palmer College of Chiropractic – Florida | | | Palmer College of Chiropractic – West | | | Parker University | | | Southern California University of Health Sciences | | |
| --- | --- | --- | --- | --- | --- | --- | --- | --- | --- | --- | --- | --- | --- | --- | --- | --- | --- | --- | --- |
| Q3: Role in Healthcare System^3^ | BASE OUTCOME | *p* | 95% CI | | *p* | 95% CI | | *p* | 95% CI | | *p* | 95% CI | | *p* | 95% CI | | *p* | 95% CI | |
| Subluxation |  | -- | -- | -- | -- | -- | -- | -- | -- | -- | -- | -- | -- | -- | -- | -- | -- | -- | -- |
| Primary Care |  | <.001 | 1.98 | 5.05 | <.001 | 1.53 | 4.12 | .55 | .45 | 4.55 | .09 | .89 | 4.52 | .01 | 1.25 | 3.50 | <.001 | 1.57 | 4.67 |
| Spine/nMSK |  | .00 | 1.30 | 2.94 | <.001 | 2.29 | 5.15 | .11 | .86 | 4.52 | <.001 | 1.78 | 6.47 | .20 | .86 | 2.04 | <.001 | 1.51 | 3.89 |
| State |  | .74 | .99 | 1.01 | <.02 | 1.00 | 1.02 | .23 | .97 | 1.01 | <.001 | .95 | .97 | <.001 | 1.02 | 1.04 | <.001 | .94 | .96 |
| Gender |  | .06 | .99 | 1.82 | .33 | .85 | 1.63 | .39 | .69 | 2.53 | .12 | .92 | 2.10 | .87 | .69 | 1.54 | .22 | .88 | 1.77 |
| Years in Practice |  | <.001 | .54 | .71 | .62 | .86 | 1.10 | <.001 | .08 | .25 | <.001 | .57 | .84 | <.001 | .48 | .67 | .37 | .82 | 1.08 |
| Survey Type |  | .93 | .66 | 1.47 | .84 | .71 | 1.52 | .74 | .53 | 2.46 | .17 | .32 | 1.23 | .20 | .44 | 1.18 | .32 | .47 | 1.28 |
| constant |  | <.001 | .21 | .59 | <.001 | .07 | .20 | .02 | .11 | .83 | .01 | .16 | .74 | <.001 | .09 | .32 | .12 | .35 | .1.13 |
| Q4: Role of SMT in Cancer Treatment^4^ | BASE OUTCOME | *p* | 95% CI | | *p* | 95% CI | | *p* | 95% CI | | *p* | 95% CI | | *p* | 95% CI | | *p* | 95% CI | |
| Innate |  | -- | -- | -- | -- | -- | -- | -- | -- | -- | -- | -- | -- | -- | -- | -- | -- | -- | -- |
| ImmuneFx |  | .01 | 1.20 | 3.39 | .01 | 1.24 | 3.80 | .53 | .41 | 5.59 | .68 | .37 | 1.91 | .78 | .64 | 1.83 | .02 | 1.21 | 5.72 |
| QoL |  | .02 | 1.12 | 3.22 | <.001 | 2.28 | 6.88 | .23 | .62 | 7.75 | .01 | 1.22 | 5.51 | .62 | .67 | 1.95 | <.001 | 2.22 | 10.35 |
| None |  | .88 | .29 | 4.20 | <.001 | 2.45 | 14.95 | .02 | 1.30 | 42.15 | .00 | 2.25 | 19.78 | .42 | .52 | 4.89 | <.001 | 4.72 | 36.29 |
| State |  | .59 | .99 | 1.01 | .01 | 1.00 | 1.02 | .22 | .97 | 1.01 | <.001 | .95 | .97 | <.001 | 1.02 | 1.03 | <.001 | .94 | .96 |
| Gender |  | .07 | .97 | 1.83 | .30 | .86 | 1.66 | .29 | .75 | 2.65 | .06 | 1.00 | 2.17 | .92 | .68 | 1.53 | .14 | .92 | 1.87 |
| Years in Practice |  | <.001 | .54 | .72 | .61 | .86 | 1.09 | <.001 | .08 | .26 | <.001 | .57 | .84 | <.001 | .49 | .68 | .69 | .84 | 1.13 |
| Survey Type |  | .74 | .62 | 1.40 | .86 | .71 | 1.52 | .59 | .57 | 2.69 | .18 | .32 | 1.24 | .20 | .44 | 1.19 | .33 | .47 | 1.29 |
| constant |  | .00 | .20 | .68 | <.001 | .05 | .20 | .05 | .06 | .98 | .11 | .21 | 1.16 | <.001 | .11 | .42 | .01 | .14 | .76 |

**Palmer**: Palmer College of Chiropractic – Davenport, Iowa

^3^Survey Question 3 labels**: nMSK:** neuromusculoskeletal conditions

^4^Survey Question 4 labels: **Innate:** Removing Interference to Innate Intelligence; **ImmuneFx:** Improving Nervous System/Immune System Function; **QoL:** Improving Pain/Quality of Life; **None:** No Role

|  | Palmer | Sherman College of Chiropractic | | | Texas Chiropractic College | | | University of Bridgeport | | | University of Western States | | |
| --- | --- | --- | --- | --- | --- | --- | --- | --- | --- | --- | --- | --- | --- |
| Q3: Role in Healthcare System^3^ | BASE OUTCOME | *p* | 95% CI | | *p* | 95% CI | | *p* | 95% CI | | *p* | 95% CI | |
| Subluxation |  | -- | -- | -- | -- | -- | -- | -- | -- | -- | -- | -- | -- |
| Primary Care |  | .04 | .21 | .96 | .00 | 2.10 | 16.56 | .01 | 1.72 | 19.24 | <.001 | 3.42 | 15.68 |
| Spine/nMSK |  | <.001 | .20 | .62 | <.001 | 2.99 | 19.16 | .01 | 1.38 | 12.28 | <.001 | 3.28 | 13.10 |
| State |  | .02 | 1.00 | 1.03 | <.001 | 1.02 | 1.04 | .01 | .96 | .99.99 | .00 | 1.01 | 1.03 |
| Gender |  | .06 | .98 | 2.43 | .97 | .56 | 1.76 | .96 | .47 | 2.05 | .27 | .84 | 1.86 |
| Years in Practice |  | .75 | .83 | 1.29 | .66 | .86 | 1.27 | <.001 | .29 | ..58 | <.001 | .57 | .80 |
| Survey Type |  | .37 | .71 | 2.52 | .69 | .62 | 2.07 | .141 | .83 | 3.64 | .58 | .52 | 1.44 |
| constant |  | <.001 | .03 | .18 | <.001 | .00 | .02 | <.001 | .02 | .28 | <.001 | .01 | .06 |
| Q4: Role of SMT in Cancer Treatment^4^ | BASE OUTCOME | *p* | 95% CI | | *p* | 95% CI | | *p* | 95% CI | | *p* | 95% CI | |
| Innate |  | -- | -- | -- | -- | -- | -- | -- | -- | -- | -- | -- | -- |
| ImmuneFx |  | .07 | .32 | 1.05 | .06 | .96 | 6.69 | .98 | 0 | . | .00 | 2.06 | 17.42 |
| QoL |  | <.001 | .09 | .45 | .01 | 1.49 | 10.17 | .98 | 0 | . | <.001 | 4.60 | 37.85 |
| None |  | .36 | .05 | 3.03 | .18 | .58 | 18.53 | .97 | 0 | . | .00 | 2.15 | 46.35 |
| State |  | .02 | 1.00 | 1.03 | <.001 | 1.02 | 1.05 | .02 | .96 | 1.00 | .00 | 1.01 | 1.03 |
| Gender |  | .06 | .98 | 2.41 | .64 | .47 | 1.58 | .83 | .51 | 2.31 | .27 | .83 | 1.89 |
| Years in Practice |  | .97 | .81 | 1.25 | .69 | .85 | 1.27 | <.001 | .32 | .62 | <.001 | .60 | .84 |
| Survey Type |  | .27 | .76 | 2.69 | .70 | .62 | 2.07 | .11 | .86 | 3.90 | .50 | .50 | 1.40 |
| constant |  | <.001 | .04 | .22 | <.001 | .00 | .03 | .97 | 0 | . | <.001 | .00 | .05 |

**Palmer**: Palmer College of Chiropractic – Davenport, Iowa

^3^Survey Question 3 labels**: nMSK:** neuromusculoskeletal conditions

^4^Survey Question 4 labels: **Innate:** Removing Interference to Innate Intelligence; **ImmuneFx:** Improving Nervous System/Immune System Function; **QoL:** Improving Pain/Quality of Life; **None:** No Role

|  | Palmer | Cleveland College of Chiropractic – Kansas City | | | Cleveland College of Chiropractic – Los Angeles | | | Life University | | | Life University West | | | Logan University | | | National University of Health Sciences | | | |
| --- | --- | --- | --- | --- | --- | --- | --- | --- | --- | --- | --- | --- | --- | --- | --- | --- | --- | --- | --- | --- |
| Q5: Vaccination | BASE OUTCOME | *p* | 95% CI | | *P* | 95% CI | | *p* | 95% CI | | *p* | 95% CI | | *p* | 95% CI | | *p* | 95% CI | | |
| Strongly agree |  | -- | -- | -- | -- | -- | -- | -- | -- | -- | -- | -- | -- | -- | -- | -- | -- | -- | -- | |
| Agree |  | .79 | .54 | 2.27 | .19 | .17 | 1.42 | .06 | .97 | 3.50 | .79 | .37 | 2.13 | .25 | .45 | 1.23 | .02 | .35 | .90 | |
| Neutral |  | .78 | .43 | 1.88 | .81 | .33 | 2.38 | .17 | .83 | 3.00 | .40 | .27 | 1.67 | .02 | .33 | .92 | <.001 | .15 | .44 | |
| Disagree |  | .42 | .35 | 1.56 | .05 | .10 | 1.01 | .04 | 1.04 | 3.74 | .18 | .21 | 1.35 | .02 | .32 | .89 | <.001 | .11 | .34 | |
| Strongly disagree |  | .10 | .23 | 1.12 | .03 | .08 | .90 | .03 | 1.06 | 3.78 | .59 | .33 | 1.90 | <.001 | .19 | .57 | <.001 | .08 | .26 | |
| State |  | .00 | .98 | .99 | <.001 | .93 | .97 | <.001 | .98 | .99 | <.001 | .94 | .97 | .11 | .99 | 1.00 | .03 | .98 | 1.00 | |
| Gender |  | .97 | .66 | 1.55 | .01 | 1.16 | 2.49 | .81 | .71 | 1.30 | .17 | .87 | 2.11 | .79 | .69 | 1.33 | .13 | .93 | 1.84 | |
| Years in Practice |  | <.001 | .62 | .86 | .26 | .90 | 1.48 | <.001 | .71 | .89 | <.001 | .58 | .83 | <.001 | .69 | .88 | .00 | 1.06 | 1.37 | |
| Survey Type |  | .38 | .77 | 1.95 | .14 | .12 | 1.34 | .90 | .73 | 1.43 | .67 | .64 | 2.02 | .42 | .81 | 1.65 | .40 | .54 | 1.28 | |
| constant |  | .44 | .34 | 1.60 | .13 | .14 | 1.30 | .39 | .39 | 1.45 | .72 | .48 | 2.90 | .12 | .89 | 2.68 | .73 | .51 | 1.60 | |
| Q6: Subluxation Detection | BASE OUTCOME | *p* | 95% CI | | *p* | 95% CI | | *p* | 95% CI | | *p* | 95% CI | | *p* | 95% CI | | *p* | 95% CI | | |
| Strongly agree |  | -- | -- | -- | -- | -- | -- | -- | -- | -- | -- | -- | -- | -- | -- | -- | -- | -- | | -- |
| Agree |  | .95 | .62 | 1.65 | .76 | .39 | 1.99 | .91 | .75 | 1.38 | .41 | .70 | 2.41 | .02 | 1.09 | 2.39 | <.001 | 1.49 | | 3.98 |
| Neutral |  | .13 | .89 | 2.59 | .04 | 1.02 | 4.94 | .87 | .67 | 1.41 | .01 | 1.27 | 4.59 | <.001 | 1.74 | 4.09 | <.001 | 2.88 | | 8.08 |
| Disagree |  | .03 | 1.08 | 3.58 | .33 | .58 | 5.07 | .56 | .54 | 1.39 | .03 | 1.09 | 5.03 | <.001 | 2.46 | 6.27 | <.001 | 3.87 | | 11.99 |
| Strongly Disagree |  | .27 | .72 | 3.19 | .52 | .40 | 6.14 | .03 | .25 | .94 | .98 | .32 | 3.19 | <.001 | 1.96 | 5.88 | <.001 | 6.66 | | 22.11 |
| State |  | .00 | .98 | .99 | <.001 | .93 | .97 | <.001 | .98 | .99 | <.001 | .94 | .97 | .04 | .99 | 1.00 | .01 | .98 | | 1.00 |
| Gender |  | .78 | .62 | 1.44 | .03 | 1.04 | 2.23 | .99 | .75 | 1.34 | .40 | .77 | 1.90 | .35 | .61 | 1.19 | .71 | .76 | | 1.50 |
| Years in Practice |  | <.001 | .63 | .87 | .20 | .92 | 1.52 | <.001 | .71 | .88 | <.001 | .57 | .86 | <.001 | .71 | .90 | <.001 | 1.15 | | 1.49 |
| Survey Type |  | .60 | .71 | 1.80 | .09 | .11 | 1.18 | .84 | .74 | 1.45 | .74 | .62 | 1.97 | .92 | .71 | 1.45 | .06 | .43 | | 1.02 |
| constant |  | .02 | .29 | .89 | <.001 | .07 | .44 | .08 | .96 | 2.07 | .14 | .31 | 1.19 | .00 | .30 | .76 | <.001 | .05 | | .16 |

**Palmer**: Palmer College of Chiropractic – Davenport, Iowa

|  | Palmer | Northwestern University of Health Sciences | | | New York Chiropractic College | | | Palmer College of Chiropractic – Florida | | | Palmer College of Chiropractic – West | | | Parker University | | | Southern California University of Health Sciences | | |
| --- | --- | --- | --- | --- | --- | --- | --- | --- | --- | --- | --- | --- | --- | --- | --- | --- | --- | --- | --- |
| Q5: Vaccination | BASE OUTCOME | *p* | 95% CI | | *p* | 95% CI | | *p* | 95% CI | | *p* | 95% CI | | *p* | 95% CI | | *p* | 95% CI | |
| Strongly agree |  | -- | -- | -- | -- | -- | -- | -- | -- | -- | -- | -- | -- | -- | -- | -- | -- | -- | -- |
| Agree |  | .39 | .44 | 1.38 | .01 | .31 | .82 | .79 | .28 | 2.63 | .16 | .30 | 1.21 | .61 | .39 | 1.76 | .01 | .25 | .78 |
| Neutral |  | .14 | .36 | 1.16 | .00 | .28 | .74 | .16 | .12 | 1.42 | .00 | .15 | .67 | .63 | .58 | 2.48 | <.001 | .18 | .57 |
| Disagree |  | .05 | .30 | .99 | <.001 | .15 | .43 | .51 | .21 | 2.17 | <.001 | .07 | .42 | .76 | .54 | 2.34 | <.001 | .13 | .44 |
| Strongly disagree |  | <.001 | .17 | .59 | <.001 | .06 | .21 | .15 | .10 | 1.40 | .00 | .12 | .56 | .63 | .39 | 1.76 | <.001 | .12 | .42 |
| State |  | .70 | .99 | 1.01 | .01 | 1.00 | 1.02 | .23 | .97 | 1.01 | <.001 | .95 | .97 | <.001 | 1.02 | 1.04 | <.001 | .94 | .96 |
| Gender |  | .02 | 1.06 | 1.97 | .14 | .93 | 1.77 | .36 | .71 | 2.66 | .08 | .96 | 2.21 | .91 | .68 | 1.53 | .08 | .97 | 1.95 |
| Years in Practice |  | <.001 | .55 | .73 | .64 | .86 | 1.10 | <.001 | .08 | .25 | <.001 | .56 | .82 | <.001 | .49 | .68 | .65 | .84 | 1.12 |
| Survey Type |  | .98 | .66 | 1.49 | .71 | .73 | 1.57 | .68 | .54 | 2.54 | .19 | .33 | 1.25 | .21 | .44 | 1.20 | .39 | .49 | 1.32 |
| constant |  | .99 | .54 | 1.87 | .22 | .40 | 1.23 | .69 | .24 | 2.54 | .05 | 1.00 | 4.54 | <.001 | .10 | .50 | <.001 | 1.73 | 5.93 |
| Q6: Subluxation Detection | BASE OUTCOME | *p* | 95% CI | | *p* | 95% CI | | *p* | 95% CI | | *P* | 95% CI | | *p* | 95% CI | | *p* | 95% CI | |
| Strongly agree |  | -- | -- | -- | -- | -- | -- | -- | -- | -- | -- | -- | -- | -- | -- | -- | -- | -- | -- |
| Agree |  | .00 | 1.21 | 2.86 | <.001 | 2.14 | 6.02 | .51 | .29 | 1.86 | .07 | .94 | 3.68 | .08 | .95 | 2.43 | .04 | 1.03 | 2.85 |
| Neutral |  | <.001 | 1.61 | 4.13 | <.001 | 3.61 | 10.67 | .79 | .44 | 2.95 | .01 | 1.27 | 5.51 | .01 | 1.19 | 3.38 | .00 | 1.37 | 4.20 |
| Disagree |  | .00 | 1.32 | 3.99 | <.001 | 6.27 | 19.48 | .52 | .51 | 3.77 | <.001 | 2.66 | 11.77 | .04 | 1.04 | 3.47 | <.001 | 3.50 | 10.98 |
| Strongly Disagree |  | .08 | .93 | 3.48 | <.001 | 5.54 | 19.61 | .83 | .26 | 2.96 | <.001 | 2.26 | 12.22 | .57 | .59 | 2.63 | <.001 | 2.09 | 8.57 |
| State |  | .58 | .99 | 1.01 | .03 | 1.00 | 1.02 | .27 | .97 | 1.01 | <.001 | .95 | .97 | <.001 | 1.02 | 1.03 | <.001 | .93 | .96 |
| Gender |  | .09 | .96 | 1.77 | .71 | .76 | 1.48 | .44 | .69 | 2.32 | .37 | .80 | 1.84 | .85 | .65 | 1.43 | .36 | .83 | 1.67 |
| Years in Practice |  | <.001 | .54 | .72 | .74 | .90 | 1.16 | <.001 | .08 | .25 | .00 | .60 | .88 | <.001 | .48 | .67 | .99 | .6 | 1.16 |
| Survey Type |  | .61 | .60 | 1.35 | .45 | .59 | 1.27 | .72 | .53 | 2.50 | .07 | .27 | 1.04 | .14 | .42 | 1.13 | .12 | .41 | 1.11 |
| constant |  | <.001 | .22 | .62 | <.001 | .04 | .12 | .11 | .20 | 1.18 | .01 | .19 | .80 | <.001 | .09 | .30 | .04 | .31 | .97 |

**Palmer**: Palmer College of Chiropractic – Davenport, Iowa

|  | Palmer | Sherman College of Chiropractic | | | Texas Chiropractic College | | | University of Bridgeport | | | University of Western States | | |
| --- | --- | --- | --- | --- | --- | --- | --- | --- | --- | --- | --- | --- | --- |
| Q5: Vaccination | BASE OUTCOME | *p* | 95% CI | | *p* | 95% CI | | *p* | 95% CI | | *p* | 95% CI | |
| Strongly agree |  | -- | -- | -- | -- | -- | -- | -- | -- | -- | -- | -- | -- |
| Agree |  | .87 | .35 | 3.49 | .27 | .31 | 1.38 | .54 | .45 | 4.68 | .02 | .28 | .90 |
| Neutral |  | .75 | .25 | 2.72 | .08 | .23 | 1.08 | .92 | .28 | 3.18 | <.001 | .13 | .48 |
| Disagree |  | .81 | .27 | 2.83 | .00 | .12 | .67 | .25 | .11 | 1.76 | <.001 | .10 | .39 |
| Strongly disagree |  | .40 | .53 | 4.86 | <.001 | .06 | .42 | .03 | .03 | .86 | <.001 | .86 | .33 |
| State |  | .05 | 1.00 | 1.03 | <.001 | 1.02 | 1.04 | .01 | .96 | .99 | .00 | 1.01 | 1.03 |
| Gender |  | .04 | 1.02 | 2.48 | .91 | .57 | 1.87 | .82 | .52 | 2.30 | .04 | 1.02 | 2.18 |
| Years in Practice |  | .71 | .84 | 1.29 | .60 | .87 | 1.28 | <.001 | .30 | .59 | <.001 | .61 | .84 |
| Survey Type |  | .31 | .74 | 2.61 | .56 | .65 | 2.20 | .13 | .85 | 3.76 | .66 | .54 | 1.48 |
| constant |  | <.001 | .01 | .15 | <.001 | .02 | .17 | .08 | .09 | 1.16 | .00 | .17 | .71 |
| Q6: Subluxation Detection | BASE OUTCOME | *p* | 95% CI | | *p* | 95% CI | | *p* | 95% CI | | *p* | 95% CI | |
| Strongly agree |  | -- | -- | -- | -- | -- | -- | -- | -- | -- | -- | -- | -- |
| Agree |  | .11 | .89 | 3.10 | .91 | .57 | 1.88 | .17 | .66 | 10.34 | .48 | .66 | 2.43 |
| Neutral |  | .34 | .68 | 3.09 | .61 | .59 | 2.43 | .01 | 1.66 | 24.53 | <.001 | 1.73 | 6.33 |
| Disagree |  | .62 | .24 | 2.33 | .21 | .76 | 3.47 | .00 | 2.01 | 31.57 | <.001 | 4.60 | 16.19 |
| Strongly Disagree |  | .48 | .13 | 2.65 | .24 | .70 | 4.15 | <.001 | 3.24 | 48.29 | <.001 | 3.50 | 14.48 |
| State |  | .03 | 1.00 | 1.03 | <.001 | 1.02 | 1.04 | .00 | .96 | 1.99 | .00 | 1.00 | 1.03 |
| Gender |  | .08 | .96 | 2.24 | .82 | .53 | 1.65 | .78 | .43 | 1.91 | .39 | .80 | 1.78 |
| Years in Practice |  | .66 | .85 | 1.30 | .57 | .87 | 1.29 | <.001 | .33 | .65 | .00 | .66 | .92 |
| Survey Type |  | .25 | .77 | 2.74 | .83 | .58 | 1.96 | .36 | .67 | 2.98 | .13 | .40 | 1.12 |
| constant |  | <.001 | .01 | .09 | <.001 | .01 | .06 | <.001 | .02 | .24 | <.001 | .02 | .09 |

**Palmer**: Palmer College of Chiropractic – Davenport, Iowa

|  | Palmer | Cleveland College of Chiropractic – Kansas City | | | Cleveland College of Chiropractic – Los Angeles | | | Life University | | | Life University West | | | Logan University | | | National University of Health Sciences | | |
| --- | --- | --- | --- | --- | --- | --- | --- | --- | --- | --- | --- | --- | --- | --- | --- | --- | --- | --- | --- |
| Q7: % of New Patient X-Rays | BASE OUTCOME | *p* | 95% CI | | *p* | 95% CI | | *p* | 95% CI | | *p* | 95% CI | | *p* | 95% CI | | *p* | 95% CI | |
| 0-20% |  | -- | -- | -- | -- | -- | -- | -- | -- | -- | -- | -- | -- | -- | -- | -- | -- | -- | -- |
| 21-40% |  | .26 | .79 | 2.35 | .38 | .63 | 3.35 | .18 | .50 | 1.14 | .85 | .47 | 1.85 | .39 | .55 | 1.26 | .49 | .76 | 1.77 |
| 41-60% |  | .58 | .64 | 2.24 | .39 | .16 | 2.02 | .65 | .72 | 1.71 | .50 | .63 | 2.56 | .93 | .62 | 1.55 | .91 | .60 | 1.58 |
| 61-80% |  | .91 | .55 | 1.95 | .83 | .44 | 2.78 | .84 | .62 | 1.48 | .22 | .25 | 1.39 | .76 | .59 | 1.46 | .06 | .35 | 1.02 |
| 81-100% |  | .31 | .47 | 1.27 | .11 | .22 | 1.17 | .67 | .67 | 1.29 | .08 | .31 | 1.06 | <.001 | .34 | .73 | <.001 | .20 | .49 |
| State |  | .00 | .98 | .99 | <.001 | .93 | .97 | <.001 | .98 | .99 | <.001 | .94 | .97 | .09 | .99 | 1.00 | .01 | .98 | 1.00 |
| Gender |  | .79 | .62 | 1.45 | .03 | 1.03 | 2.24 | .97 | .75 | 1.35 | .28 | .83 | 1.92 | .36 | .62 | 1.19 | .79 | .74 | 1.48 |
| Years in Practice |  | <.001 | .62 | .85 | .29 | .89 | 1.47 | <.001 | .72 | .90 | <.001 | .58 | .85 | <.001 | .69 | .87 | .00 | 1.06 | 1.36 |
| Survey Type |  | .99 | .60 | 1.65 | .05 | .08 | 1.00 | .71 | .74 | .1.54 | .99 | .53 | 1.86 | .82 | .71 | 1.53 | .04 | .39 | .97 |
| constant |  | .11 | .37 | 1.10 | .00 | .11 | .65 | .11 | .93 | 2.02 | .81 | .58 | 2.01 | .51 | .76 | 1.73 | .00 | .28 | .73 |

**Palmer**: Palmer College of Chiropractic – Davenport, Iowa

|  | Palmer | Northwestern University of Health Sciences | | | New York Chiropractic College | | | Palmer College of Chiropractic – Florida | | | Palmer College of Chiropractic – West | | | Parker University | | | Southern California University of Health Sciences | | |
| --- | --- | --- | --- | --- | --- | --- | --- | --- | --- | --- | --- | --- | --- | --- | --- | --- | --- | --- | --- |
| Q7: % of New Patient X-Rays | BASE OUTCOME | *p* | 95% CI | | *p* | 95% CI | | *p* | 95% CI | | *p* | 95% CI | | *p* | 95% CI | | *p* | 95% CI | |
| 0-20% |  | -- | -- | -- | -- | -- | -- | -- | -- | -- | -- | -- | -- | -- | -- | -- | -- | -- | -- |
| 21-40% |  | .47 | .53 | 1.33 | .74 | .72 | 1.61 | .10 | .11 | 1.22 | .68 | .62 | 2.07 | .79 | .63 | 1.84 | .98 | .59 | 1.66 |
| 41-60% |  | .13 | .37 | 1.13 | .57 | .54 | 1.41 | .23 | .08 | 1.83 | .04 | .14 | .97 | .56 | .42 | 1.61 | .23 | 36 | 1.28 |
| 61-80% |  | .21 | .41 | 1.21 | .30 | .48 | 1.25 | .67 | .45 | 3.47 | .65 | .43 | 1.68 | .92 | .56 | 1.89 | .89 | .61 | 1.78 |
| 81-100% |  | <.001 | .26 | .62 | <.001 | .17 | .42 | .82 | .40 | 2.07 | <.001 | .10 | .44 | .40 | .52 | 1.30 | .01 | .32 | .82 |
| State |  | .56 | .99 | 1.01 | .02 | 1.00 | 1.02 | .30 | .97 | 1.01 | <.001 | .95 | .97 | <.001 | 1.02 | 1.03 | <.001 | .94 | .96 |
| Gender |  | .11 | .96 | 1.74 | .85 | .74 | 1.44 | .39 | .71 | 2.46 | .41 | .78 | 1.82 | .93 | .66 | 1.46 | 40 | .82 | 1.66 |
| Years in Practice |  | <.001 | .55 | .73 | .59 | .86 | 1.09 | <.001 | .08 | .25 | <.001 | .57 | .83 | <.001 | .49 | .67 | .47 | .82 | 1.10 |
| Survey Type |  | .65 | .58 | 1.40 | .31 | .54 | 1.22 | .23 | .71 | 4.00 | .04 | .23 | .95 | .14 | .39 | 1.14 | .15 | .40 | 1.15 |
| constant |  | .56 | .55 | 1.38 | <.001 | .25 | .64 | .14 | .22 | 1.22 | .32 | .75 | 2.42 | <.001 | .14 | .46 | .08 | .95 | 2.57 |

**Palmer**: Palmer College of Chiropractic – Davenport, Iowa

|  | Palmer | Sherman College of Chiropractic | | | Texas Chiropractic College | | | University of Bridgeport | | | University of Western States | | |
| --- | --- | --- | --- | --- | --- | --- | --- | --- | --- | --- | --- | --- | --- |
| Q7: % of New Patient X-Rays | BASE OUTCOME | *p* | 95% CI | | *p* | 95% CI | | *p* | 95% CI | | *p* | 95% CI | |
| 0-20% |  | -- | -- | -- | -- | -- | -- | -- | -- | -- | -- | -- | -- |
| 21-40% |  | .11 | .19 | 1.19 | .69 | .57 | 2.32 | .11 | .85 | 4.80 | .69 | .67 | 1.86 |
| 41-60% |  | .39 | .65 | 3.09 | .92 | .46 | 2.35 | .42 | .55 | 4.13 | .00 | .08 | .58 |
| 61-80% |  | .85 | .39 | 2.17 | .88 | .42 | 2.11 | .32 | .10 | 2.11 | .22 | .33 | 1.28 |
| 81-100% |  | .97 | .51 | 1.90 | .79 | .50 | 1.69 | .02 | .01 | .70 | <.001 | .17 | .55 |
| State |  | .03 | 1.00 | 1.03 | <.001 | 1.02 | 1.04 | .01 | .96 | .99 | .00 | 1.01 | 1.03 |
| Gender |  | .04 | 1.3 | 2.30 | .79 | .52 | 1.64 | .71 | .41 | 1.83 | .38 | .80 | 1.76 |
| Years in Practice |  | .61 | .85 | 1.31 | .69 | .86 | 1.26 | <.001 | .29 | .58 | <.001 | .61 | .84 |
| Survey Type |  | .14 | .84 | 3.35 | .93 | .53 | 2.00 | .84 | .41 | 2.08 | .21 | .40 | 1.23 |
| constant |  | <.001 | .02 | .11 | <.001 | .01 | .07 | .03 | .14 | .91 | <.001 | .10 | .33 |

**Palmer**: Palmer College of Chiropractic – Davenport, Iowa
