# Supplementary material for: Associations between demographics and clinical ideology, beliefs, and practice patterns: a secondary analysis of a survey of randomly sampled United States chiropractors
Source: BMC Complement Med Ther. 2023 Nov 9;23:404. doi: 10.1186/s12906-023-04225-z (PMC10634061; doi:10.1186/s12906-023-04225-z)
Supplement: Supplementary file 1 — Additional file 1: Appendix 1A. Multinomial logistic regression models 95% confidence interval output for the relative risk ratio given the other predictors are in the model: chiropractic degree program of graduation. Appendix 1B. Table Multinomial logistic regression models 95% confidence interval output for the relative risk ratio given the other predictors are in the model: years since of chiropractic degree completion. Appendix 1C. Multinomial logistic regression models 95% confidence interval output for the relative risk ratio given the other predictors are in the model: primary practice location (US Census region). [file 12906_2023_4225_MOESM1_ESM.zip › Appendix_1B_Years_since_degree_table_v1_7.31.2023.docx]

Appendix 1B. Table Multinomial logistic regression models 95% confidence interval output for the relative risk ratio given the other predictors are in the model: years since of chiropractic degree completion

|  | 1-10 years | 11-20 years | | | 21-30 years | | | 31-40 years | | | 41 or more years | | |
| --- | --- | --- | --- | --- | --- | --- | --- | --- | --- | --- | --- | --- | --- |
| Q1: Scope of Examination^1^ | BASE OUTCOME | *p* | 95% CI | | *p* | 95% CI | | *p* | 95% CI | | *p* | 95% CI | |
| SA |  | -- | -- | -- | -- | -- | -- | -- | -- | -- | -- | -- | -- |
| SA>DDx |  | .16 | .29 | 1.23 | .61 | .41 | 1.70 | .06 | .98 | 4.13 | .73 | .25 | 2.66 |
| SA+DDx |  | .31 | .57 | 1.20 | .98 | .68 | 1.48 | .06 | .98 | 2.31 | .13 | .42 | 1.12 |
| DDx>SA |  | .95 | .44 | 2.39 | .85 | .38 | 2.25 | .77 | .43 | 3.12 | .26 | .03 | 2.50 |
| DDX |  | .003 | .29 | .78 | .003 | .27 | .78 | .39 | .46 | 1.35 | <.001 | .09 | .44 |
| State |  | .83 | 1.00 | 1.01 | .96 | .99 | 1.01 | .03 | .99 | 1.00 | .32 | .99 | 1.00 |
| Gender |  | .11 | .71 | 1.04 | <.001 | .45 | .71 | <.001 | .31 | .51 | <.001 | .07 | .22 |
| Chiropractic College |  | .88 | .98 | 1.03 | .16 | .96 | 1.01 | .78 | .98 | 1.03 | .27 | .99 | 1.06 |
| Survey Type |  | .001 | .44 | .80 | <.001 | .42 | .78 | <.001 | .35 | .67 | <.001 | .16 | .52 |
| constant |  | .001 | 1.34 | 3.42 | <.001 | 1.59 | 4.16 | .04 | 1.03 | 2.88 | .88 | .55 | 2.00 |
| Q2: Conditions treated^2^ | BASE OUTCOME | *p* | 95% CI | | *p* | 95% CI | | *p* | 95% CI | | *p* | 95% CI | |
| VS |  | -- | -- | -- | -- | -- | -- | -- | -- | -- | -- | -- | -- |
| Broad |  | .69 | .77 | 1.50 | .32 | .85 | 1.66 | .01 | 1.09 | 2.20 | .13 | .90 | 2.31 |
| Somatovisc |  | .52 | .64 | 2.38 | .13 | .86 | 3.08 | .001 | 1.49 | 5.21 | .03 | 1.06 | 5.16 |
| MSKsub |  | .86 | .60 | 1.52 | .72 | .68 | 1.74 | .15 | .88 | 2.29 | .94 | .52 | 2.01 |
| MSKgen |  | .85 | .69 | 1.58 | .72 | .60 | 1.42 | .41 | .77 | 1.86 | .26 | .79 | 2.45 |
| nMSK |  | .20 | .59 | 1.12 | .07 | .54 | 1.02 | .13 | .55 | 1.08 | <.001 | .24 | .67 |
| State |  | .88 | 1.00 | 1.01 | .91 | .99 | 1.01 | .02 | .99 | 1.00 | .31 | .99 | 1.00 |
| Gender |  | .16 | .72 | 1.05 | <.001 | .44 | .69 | <.001 | .32 | .52 | <.001 | .07 | .21 |
| Chiropractic College |  | .76 | .98 | 1.03 | .17 | .96 | 1.01 | .82 | .98 | 1.03 | .28 | .98 | 1.06 |
| Survey Type |  | <.001 | .43 | .74 | <.001 | .42 | .72 | <.001 | .39 | .68 | <.001 | .17 | .47 |
| constant |  | .01 | 1.18 | 2.63 | <.001 | 1.68 | 3.76 | <.001 | 1.40 | 3.22 | .28 | .41 | 1.30 |

^1^ Survey Question 1 labels: **SA:** Spinal analysis to detect subluxation only; **SA>DDx:** Focus on Spinal analysis, sometimes includes differential diagnosis; **SA+DDx:** Equal focus on spinal analysis to detect subluxation and differential diagnosis; **DDx>SA:** Focus on differential diagnosis, sometimes includes spinal analysis; **DDx:** Differential Diagnosis only

^2^ Survey Question 2 labels: **VS:** Vertebral Subluxation as an Encumbrance to Health; **Broad:** Broad Spectrum of Health Concerns Including Lifestyle and Wellness Issues; **Somatovisc:** Biomechanical and Organic/Visceral Conditions; **MSKsub:** Vertebral Subluxation as a Musculoskeletal Condition; **MSKgen:** General and Biomechanical Conditions; **nMSK:** Neuromusculoskeletal Conditions

|  | 1-10 years | 11-20 years | | | | 21-30 years | | | 31-40 years | | | | 41 or more years | | | |
| --- | --- | --- | --- | --- | --- | --- | --- | --- | --- | --- | --- | --- | --- | --- | --- | --- |
| Q3: Role in healthcare system | BASE OUTCOME | *p* | 95% CI | | | *p* | 95% CI | | *p* | 95% CI | | | *p* | 95% CI | | |
| Subluxation |  | -- | -- | -- | | -- | -- | -- | -- | -- | -- | | -- | -- | -- | |
| Primary Care |  | .87 | .69 | 1.37 | | .25 | .87 | 1.72 | .001 | 1.25 | 2.51 | | .04 | 1.02 | 2.64 | |
| Spine/NMSK |  | .10 | .61 | 1.04 | | .18 | .63 | 1.09 | .87 | .73 | 1.30 | | .33 | .54 | 1.23 | |
| State |  | .71 | 1.00 | 1.01 | | .79 | 1.00 | 1.01 | .03 | .99 | 1.00 | | .35 | .99 | 1.00 | |
| Gender |  | .17 | .73 | 1.06 | | <.001 | .46 | .72 | <.001 | .31 | .51 | | <.001 | .07 | .22 | |
| Chiropractic College |  | .96 | .98 | 1.02 | | .10 | .96 | 1.00 | .90 | .97 | 1.02 | | .43 | .98 | 1.05 | |
| Survey Type |  | <.001 | .44 | .75 | | <.001 | .43 | .73 | <.001 | .39 | .69 | | <.001 | .17 | .47 | |
| constant |  | .001 | 1.31 | 2.84 | | <.001 | 1.76 | 3.84 | <.001 | 1.46 | 3.28 | | .25 | .41 | 1.26 | |
| Q4: Role of SMT in Cancer Treatment^3^ | BASE OUTCOME | *p* | 95% CI | | | *p* | 95% CI | | *p* | 95% CI | | | *p* | 95% CI | | |
| Innate |  | -- | -- | | -- | -- | -- | -- | -- | -- | | -- | -- | -- | | -- |
| ImmuneFx |  | .51 | .60 | | 1.29 | .57 | .76 | 1.65 | .81 | .71 | | 1.57 | .74 | .55 | | 1.54 |
| QoL |  | .01 | .40 | | .85 | .02 | .44 | .94 | .08 | .48 | | 1.05 | .003 | .27 | | .76 |
| None |  | .55 | .43 | | 1.57 | .82 | .48 | 1.80 | .41 | .38 | | 1.48 | .92 | .42 | | 2.21 |
| State |  | .77 | 1.00 | | 1.01 | .76 | 1.00 | 1.01 | .01 | .99 | | 1.00 | .32 | .99 | | 1.00 |
| Gender |  | .11 | .70 | | 1.03 | <.001 | .45 | .71 | <.001 | .30 | | .49 | <.001 | .07 | | .21 |
| Chiropractic College |  | .71 | .98 | | 1.03 | .32 | .96 | 1.01 | .93 | .98 | | 1.03 | .23 | .99 | | 1.06 |
| Survey Type |  | <.001 | .44 | | .74 | <.001 | .43 | .73 | <.001 | .38 | | .67 | <.001 | .17 | | .47 |
| constant |  | .001 | 1.41 | | 3.52 | <.001 | 1.63 | 4.15 | <.001 | 1.81 | | 4.69 | .92 | .51 | | 1.84 |
| Q5: Vaccination | BASE OUTCOME | *p* | 95% CI | | | *p* | 95% CI | | *p* | 95% CI | | | *p* | 95% CI | | |
| Strongly agree |  | -- | -- | | -- | -- | -- | -- | -- | -- | | -- | -- | -- | | -- |
| Agree |  | .04 | 1.02 | | 2.14 | .03 | 1.05 | 2.21 | .23 | .87 | | 1.78 | 0.004 | 1.30 | | 3.91 |
| Neutral |  | .08 | .96 | | 2.04 | .15 | .91 | 1.94 | .68 | .64 | | 1.34 | .20 | .82 | | 2.62 |
| Disagree |  | .003 | 1.22 | | 2.67 | .01 | 1.17 | 2.58 | .52 | .77 | | 1.68 | .06 | .97 | | 3.26 |
| Strongly disagree |  | <.001 | 1.51 | | 3.45 | <.001 | 1.61 | 3.69 | .03 | 1.05 | | 2.37 | .001 | 1.48 | | 5.03 |
| State |  | .97 | .99 | | 1.01 | .95 | .99 | 1.01 | .01 | .99 | | 1.00 | .23 | .99 | | 1.00 |
| Gender |  | .05 | .68 | | 1.00 | <.001 | .44 | .69 | <.001 | .32 | | .52 | <.001 | .08 | | .22 |
| Chiropractic College |  | .71 | .98 | | 1.03 | .20 | .96 | 1.01 | .94 | .98 | | 1.03 | .27 | .99 | | 1.06 |
| Survey Type |  | <.001 | .43 | | .73 | <.001 | .41 | .71 | <.001 | .38 | | .66 | <.001 | .17 | | .45 |
| constant |  | .67 | .70 | | 1.72 | .05 | 1.01 | 2.46 | .001 | 1.40 | | 3.38 | .01 | .20 | | .75 |

^3^ Survey Question 4 labels: **Innate:** Removing Interference to Innate Intelligence; **ImmuneFx:** Improving Nervous System/Immune System Function; **QoL:** Improving Pain/Quality of Life; **None:** No Role

|  | 1-10 years | 11-20 years | | | 21-30 years | | | 31-40 years | | | 41 or more years | | |
| --- | --- | --- | --- | --- | --- | --- | --- | --- | --- | --- | --- | --- | --- |
| Q6: Subluxation Detection | BASE OUTCOME | *p* | 95% CI | | *p* | 95% CI | | *p* | 95% CI | | *p* | 95% CI | |
| Strongly agree |  | -- | -- | -- | -- | -- | -- | -- | -- | -- | -- | -- | -- |
| Agree |  | .90 | .74 | 1.40 | .05 | .99 | 1.88 | .08 | .97 | 1.83 | .09 | .94 | 2.20 |
| Neutral |  | .50 | .64 | 1.24 | .56 | .79 | 1.55 | .53 | .63 | 1.27 | .77 | .67 | 1.71 |
| Disagree |  | .03 | .48 | .95 | .04 | .48 | .98 | .02 | .45 | .92 | .01 | .27 | .80 |
| Strongly Disagree |  | <.001 | .34 | .72 | <.001 | .30 | .66 | <.001 | .25 | .56 | <.001 | .04 | .27 |
| State |  | .68 | 1.00 | 1.01 | .62 | 1.00 | 1.01 | .04 | .99 | 1.00 | .99 | .99 | 1.00 |
| Gender |  | .18 | .72 | 1.06 | <.001 | .45 | .71 | <.001 | .32 | .52 | .08 | .08 | .23 |
| Chiropractic College |  | .59 | .98 | 1.03 | .29 | .96 | 1.01 | .50 | .98 | 1.03 | .99 | .99 | 1.06 |
| Survey Type |  | <.001 | .47 | .79 | <.001 | .45 | .78 | <.001 | .41 | .73 | .20 | .20 | .52 |
| constant |  | .001 | 1.28 | 2.74 | <.001 | 1.55 | 3.36 | <.001 | 1.67 | 3.63 | .40 | .40 | 1.20 |
| Q7: % of New Patient X-Rays | BASE OUTCOME | *p* | 95% CI | | *p* | 95% CI | | *p* | 95% CI | | *p* | 95% CI | |
| 0-20% |  | -- | -- | -- | -- | -- | -- | -- | -- | -- | -- | -- | -- |
| 21-40% |  | .49 | .81 | 1.54 | .03 | 1.04 | 1.96 | .18 | .90 | 1.73 | .10 | .92 | 2.36 |
| 41-60% |  | .32 | .82 | 1.80 | .03 | .105 | 2.27 | .02 | 1.07 | 2.33 | .15 | .86 | 2.64 |
| 61-80% |  | .33 | .83 | 1.77 | .17 | .89 | 1.92 | .01 | 1.16 | 2.46 | .15 | .86 | 2.52 |
| 81-100% |  | .42 | .84 | 1.52 | .45 | .83 | 1.51 | .58 | .67 | 1.25 | .21 | .86 | 1.97 |
| State |  | .80 | 1.00 | 1.01 | .95 | .99 | 1.01 | .03 | .99 | 1.00 | .22 | .99 | 1.00 |
| Gender |  | .18 | .73 | 1.06 | <.001 | .47 | .73 | <.001 | .33 | .53 | <.001 | .08 | .24 |
| Chiropractic College |  | .96 | .98 | 1.02 | .11 | .96 | 1.00 | .99 | .98 | 1.03 | .35 | .98 | 1.05 |
| Survey Type |  | <.001 | .41 | .74 | <.001 | .36 | .65 | <.001 | .33 | .50 | <.001 | .15 | .42 |
| constant |  | .01 | 1.10 | 2.25 | <.001 | 1.49 | 3.05 | <.001 | 1.51 | 3.15 | .04 | .34 | .99 |
