# Supplementary material for: Associations between demographics and clinical ideology, beliefs, and practice patterns: a secondary analysis of a survey of randomly sampled United States chiropractors
Source: BMC Complement Med Ther. 2023 Nov 9;23:404. doi: 10.1186/s12906-023-04225-z (PMC10634061; doi:10.1186/s12906-023-04225-z)
Supplement: Supplementary file 1 — Additional file 1: Appendix 1A. Multinomial logistic regression models 95% confidence interval output for the relative risk ratio given the other predictors are in the model: chiropractic degree program of graduation. Appendix 1B. Table Multinomial logistic regression models 95% confidence interval output for the relative risk ratio given the other predictors are in the model: years since of chiropractic degree completion. Appendix 1C. Multinomial logistic regression models 95% confidence interval output for the relative risk ratio given the other predictors are in the model: primary practice location (US Census region). [file 12906_2023_4225_MOESM1_ESM.zip › Appendix_1C_Region_table_v1_7.31.2023.docx]

Appendix 1C. Multinomial logistic regression models 95% confidence interval output for the relative risk ratio given the other predictors are in the model: primary practice location (US Census region)

|  | Northeast | Midwest | | | South | | | West | | | |
| --- | --- | --- | --- | --- | --- | --- | --- | --- | --- | --- | --- |
| Q1: Scope of Examination^1^ | BASE OUTCOME | *p* | 95% CI | | *p* | 95% CI | | *p* | 95% CI | | |
| SA |  | -- | -- | -- | -- | -- | -- | -- | -- | -- | |
| SA>DDx |  | .74 | .56 | 2.28 | .94 | .50 | 2.11 | .19 | .80 | 3.19 | |
| SA+DDx |  | .16 | .90 | 1.84 | .23 | .87 | 1.79 | .20 | .88 | 1.81 | |
| DDx>SA |  | .29 | .23 | 1.54 | .44 | .28 | 1.72 | .38 | .63 | 3.30 | |
| DDX |  | .39 | .74 | 2.12 | .19 | .85 | 2.38 | .17 | .86 | 2.40 | |
| Gender |  | .20 | .93 | 1.46 | .94 | .79 | 1.29 | .27 | .91 | 1.43 | |
| Chiropractic College |  | <.001 | .91 | .96 | .60 | .98 | 1.03 | <.001 | 1.07 | 1.12 | |
| Years in Practice |  | <.001 | .67 | .80 | <.001 | .69 | .82 | .001 | .80 | .95 | |
| Survey Type |  | .04 | .52 | .98 | .09 | .56 | 1.05 | .02 | .50 | .94 | |
| constant |  | <.001 | 2.77 | 6.90 | .002 | 1.32 | 3.36 | .02 | .35 | .90 | |
| Q2: Conditions treated^2^ | BASE OUTCOME | *p* | 95% CI | | *p* | 95% CI | | *p* | 95% CI | | |
| VS |  | -- | -- | -- | -- | -- | -- | -- | -- | | -- |
| Broad |  | .07 | .97 | 1.86 | .30 | .86 | 1.64 | .27 | .87 | | 1.66 |
| Somatovisc |  | .01 | 1.22 | 3.63 | .11 | .90 | 2.80 | .41 | .71 | | 2.28 |
| MSKsub |  | .50 | .75 | 1.79 | .57 | .56 | 1.37 | .73 | .59 | | 1.45 |
| MSKgen |  | .85 | .69 | 1.56 | .92 | .68 | 1.53 | .70 | .72 | | 1.62 |
| nMSK |  | .11 | .56 | 1.06 | .23 | .60 | 1.13 | .62 | .79 | | 1.49 |
| Gender |  | .46 | .87 | 1.37 | .62 | .74 | 1.20 | ..52 | .86 | | 1.35 |
| Chiropractic College |  | <.001 | .92 | .96 | .40 | .97 | 1.04 | <.001 | 1.07 | | 1.12 |
| Years in Practice |  | <.001 | .65 | .77 | <.001 | .68 | .81 | <.001 | .79 | | .93 |
| Survey Type |  | .01 | .52 | .90 | .02 | .55 | .96 | .04 | .58 | | .99 |
| constant |  | <.001 | 3.59 | 7.96 | <.001 | 1.7 | 3.91 | .04 | .42 | | .97 |

^1^ Survey Question 1 labels: **SA:** Spinal analysis to detect subluxation only; **SA>DDx:** Focus on Spinal analysis, sometimes includes differential diagnosis; **SA+DDx:** Equal focus on spinal analysis to detect subluxation and differential diagnosis; **DDx>SA:** Focus on differential diagnosis, sometimes includes spinal analysis; **DDx:** Differential Diagnosis only

^2^ Survey Question 2 labels: **VS:** Vertebral Subluxation as an Encumbrance to Health; **Broad:** Broad Spectrum of Health Concerns Including Lifestyle and Wellness Issues; **Somatovisc:** Biomechanical and Organic/Visceral Conditions; **MSKsub:** Vertebral Subluxation as a Musculoskeletal Condition; **MSKgen:** General and Biomechanical Conditions; **nMSK:** Neuromusculoskeletal Conditions

|  | Northeast | Midwest | | | | South | | | | West | | | | |
| --- | --- | --- | --- | --- | --- | --- | --- | --- | --- | --- | --- | --- | --- | --- |
| Q3: Best role for chiropractic | BASE OUTCOME | *p* | 95% CI | | | *p* | 95% CI | | | *p* | 95% CI | | | |
| Subluxation |  | -- | -- | -- | | -- | -- | | -- | -- | -- | -- | | |
| Primary Care |  | <.001 | 1.30 | 2.51 | | .001 | 1.29 | | 2.51 | .03 | 1.04 | 2.02 | | |
| Neuromusculoskeletal/Spine |  | .86 | .75 | 1.27 | | .45 | .85 | | 1.45 | .47 | .85 | 1.43 | | |
| Gender |  | .26 | .91 | 1.44 | | .97 | .79 | | 1.28 | .30 | .90 | 1.43 | | |
| Chiropractic College |  | <.001 | .91 | .96 | | .43 | .99 | | 1.03 | <.001 | 1.07 | 1.12 | | |
| Years in Practice |  | <.001 | .66 | .79 | | <.001 | .68 | | .81 | .001 | .79 | .94 | | |
| Survey Type |  | .003 | .50 | .87 | | .02 | .54 | | .94 | .03 | .57 | .97 | | |
| constant |  | <.001 | 3.48 | 7.51 | | <.001 | 1.45 | | 3.20 | .02 | .42 | .93 | | |
| Q4: Role of SMT in Cancer Treatment^3^ | BASE OUTCOME | *p* | 95% CI | | | *p* | 95% CI | | | *p* | 95% CI | | | |
| Innate |  | -- | -- | | -- | -- | -- | -- | | -- | -- | | -- | |
| ImmuneFx |  | .37 | .83 | | 1.66 | .33 | .84 | 1.69 | | .39 | .82 | | 1.67 | |
| QoL |  | .90 | .69 | | 1.38 | .68 | .65 | 1.31 | | .40 | .82 | | 1.65 | |
| None |  | .29 | .37 | | 1.35 | .94 | .53 | 1.80 | | .18 | .83 | | 2.64 | |
| Gender |  | .27 | .91 | | 1.43 | .99 | .79 | 1.27 | | .35 | .89 | | 1.40 | |
| Chiropractic College |  | <.001 | .92 | | .96 | .38 | .99 | 1.04 | | <.001 | 1.07 | | 1.12 | |
| Years in Practice |  | <.001 | .67 | | .80 | <.001 | .69 | .83 | | .001 | .80 | | .94 | |
| Survey Type |  | .002 | .50 | | .86 | .01 | .53 | .93 | | .03 | .57 | | .97 | |
| constant |  | <.001 | 3.24 | | 7.84 | <.001 | 1.50 | 3.72 | | .03 | .38 | | .94 | |
| Q5: Vaccination | BASE OUTCOME | *p* | 95% CI | | | *p* | 95% CI | | | *p* | 95% CI | | | |
| Strongly agree |  | -- | -- | | -- | -- | -- | -- | | -- | -- | | | -- |
| Agree |  | .002 | 1.25 | | 2.60 | .003 | 1.20 | 2.49 | | .88 | .70 | | | 1.37 |
| Neutral |  | .02 | 1.08 | | 2.30 | .16 | .90 | 1.93 | | .88 | .72 | | | 1.46 |
| Disagree |  | .02 | 1.10 | | 2.42 | <.001 | 1.36 | 2.97 | | .41 | .81 | | | 1.68 |
| Strongly disagree |  | .002 | 1.25 | | 2.77 | .01 | 1.16 | 2.57 | | .44 | .80 | | | 1.67 |
| Gender |  | .27 | .91 | | 1.43 | .85 | .77 | 1.24 | | .29 | .90 | | | 1.42 |
| Chiropractic College |  | <.001 | .92 | | .96 | .38 | .99 | 1.04 | | <.001 | 1.07 | | | 1.12 |
| Years in Practice |  | <.001 | .67 | | .80 | <.001 | .69 | .82 | | .002 | .80 | | | .95 |
| Survey Type |  | .002 | .50 | | .86 | .01 | .53 | .91 | | .02 | .55 | | | .95 |
| constant |  | <.001 | 2.10 | | 5.11 | .04 | 1.01 | 2.49 | | .03 | .40 | | | .96 |

^3^ Survey Question 4 labels: **Innate:** Removing Interference to Innate Intelligence; **ImmuneFx:** Improving Nervous System/Immune System Function; **QoL:** Improving Pain/Quality of Life; **None:** No Role

|  | Northeast | Midwest | | | South | | | West | | | |
| --- | --- | --- | --- | --- | --- | --- | --- | --- | --- | --- | --- |
| Q6: Subluxation Detection | BASE OUTCOME | *p* | 95% CI | | *p* | 95% CI | | *p* | 95% CI | | |
| Strongly agree |  | -- | -- | -- | -- | -- | -- | -- | -- | -- | |
| Agree |  | .19 | .61 | 1.10 | .01 | .50 | .90 | .01 | .50 | .91 | |
| Neutral |  | .28 | .60 | 1.16 | .001 | .40 | .78 | .39 | .63 | 1.20 | |
| Disagree |  | .01 | .44 | .89 | <.001 | .31 | .63 | .11 | .54 | 1.07 | |
| Strongly Disagree |  | .04 | .44 | .98 | <.001 | .23 | .54 | .08 | .47 | 1.04 | |
| Gender |  | .17 | .93 | 1.47 | .90 | .80 | 1.29 | .26 | .91 | 1.43 | |
| Chiropractic College |  | <.001 | .92 | .96 | .26 | .99 | 1.04 | <.001 | 1.07 | 1.12 | |
| Years in Practice |  | <.001 | .66 | .79 | <.001 | .67 | .80 | .001 | .79 | .94 | |
| Survey Type |  | .004 | .51 | .88 | .04 | .57 | .99 | .03 | .57 | .98 | |
| constant |  | <.001 | 4.48 | 9.79 | <.001 | 2.67 | 5.88 | .43 | .56 | 1.27 | |
| Q7: % of New Patient X-Rays | BASE OUTCOME | *p* | 95% CI | | *p* | 95% CI | | *p* | 95% CI | | |
| 0-20% |  | -- | -- | -- | -- | -- | -- | -- | -- | | -- |
| 21-40% |  | .03 | .53 | .96 | .34 | .62 | 1.18 | .60 | .69 | | 1.24 |
| 41-60% |  | .12 | .52 | 1.08 | .06 | .98 | 2.00 | .56 | .63 | | 1.28 |
| 61-80% |  | .94 | .69 | 1.41 | .01 | 1.12 | 2.29 | .64 | .76 | | 1.55 |
| 81-100% |  | .07 | .98 | 1.81 | <.001 | 2.07 | 3.83 | .19 | .90 | | 1.68 |
| Gender |  | .14 | .95 | 1.50 | .47 | .86 | 1.40 | .18 | .93 | | 1.47 |
| Chiropractic College |  | <.001 | .92 | .96 | .35 | .99 | 1.04 | <.001 | 1.07 | | 1.12 |
| Years in Practice |  | <.001 | .67 | .80 | <.001 | .68 | .82 | .001 | .79 | | .94 |
| Survey Type |  | .11 | .59 | 1.05 | .41 | .65 | 1.19 | .09 | .58 | | 1.04 |
| constant |  | <.001 | 3.79 | 7.72 | .003 | 1.21 | 2.56 | .03 | .46 | | .97 |
