# Supplementary material for: Associations between demographics and clinical ideology, beliefs, and practice patterns: a secondary analysis of a survey of randomly sampled United States chiropractors
Source: BMC Complement Med Ther. 2023 Nov 9;23:404. doi: 10.1186/s12906-023-04225-z (PMC10634061; doi:10.1186/s12906-023-04225-z)
Supplement: Supplementary file 2 — Additional file 2: Appendix 2A. Association between years since completion of chiropractic degree and ideologies, beliefs, and practice patterns. Appendix 2B. Association between region of primary practice location and ideologies, beliefs, and practice patterns. [file 12906_2023_4225_MOESM2_ESM.zip › Appendix_2A_Years_bargraphs_10.26.2023_ESM.docx]

Appendix 2A: Association between years since completion of chiropractic degree and ideologies, beliefs, and practice patterns

| 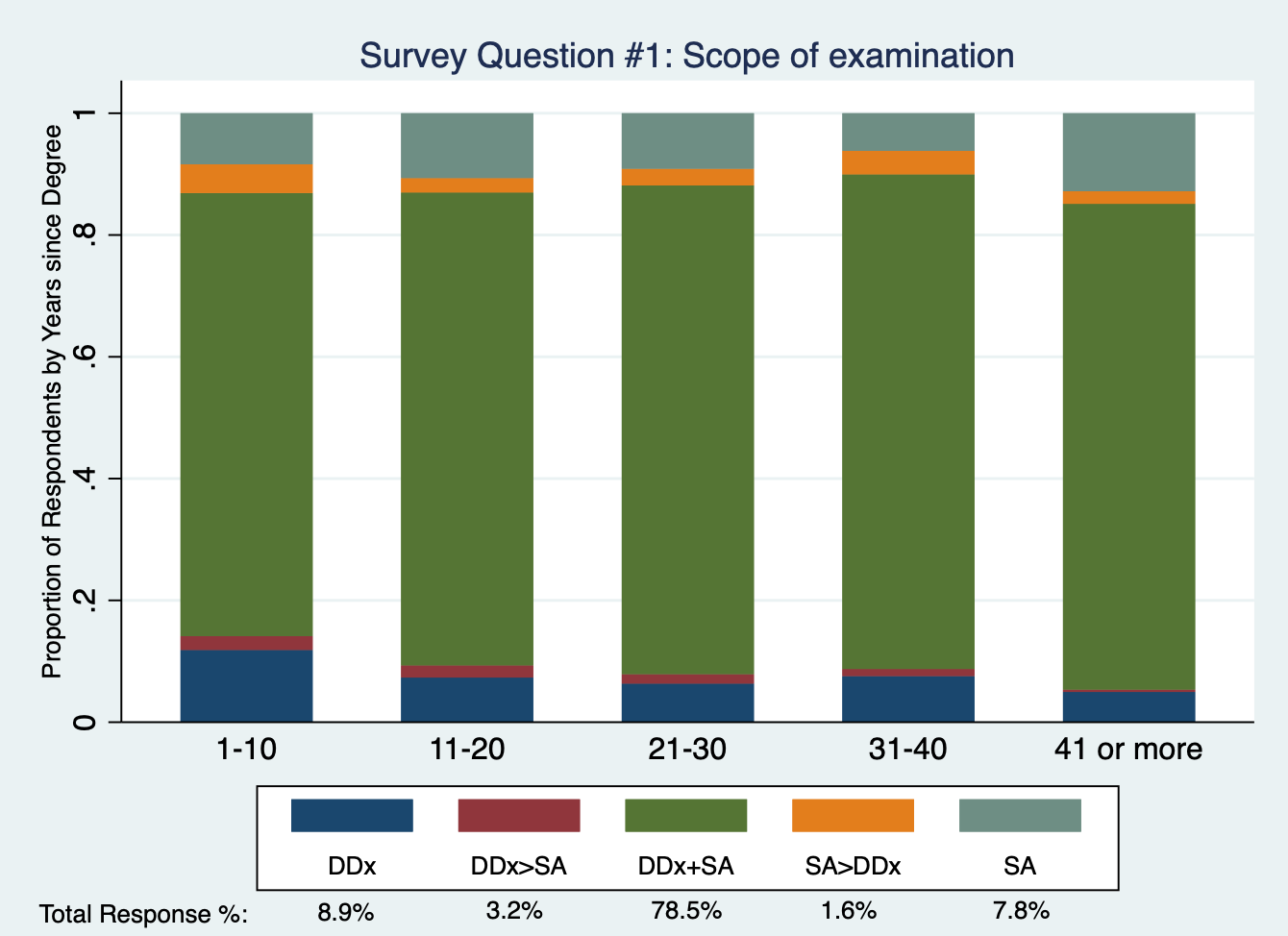 | 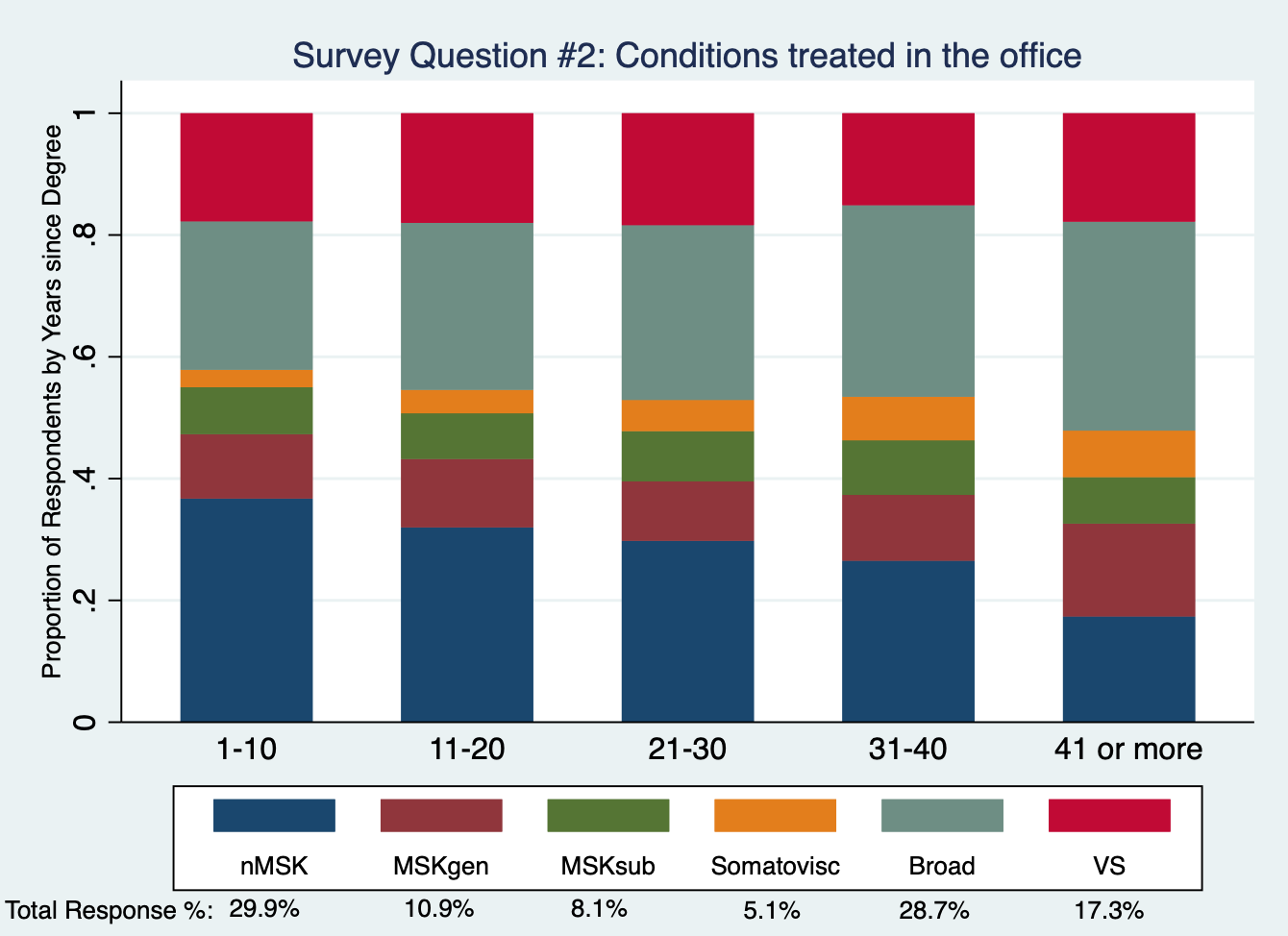 |
| --- | --- |
| 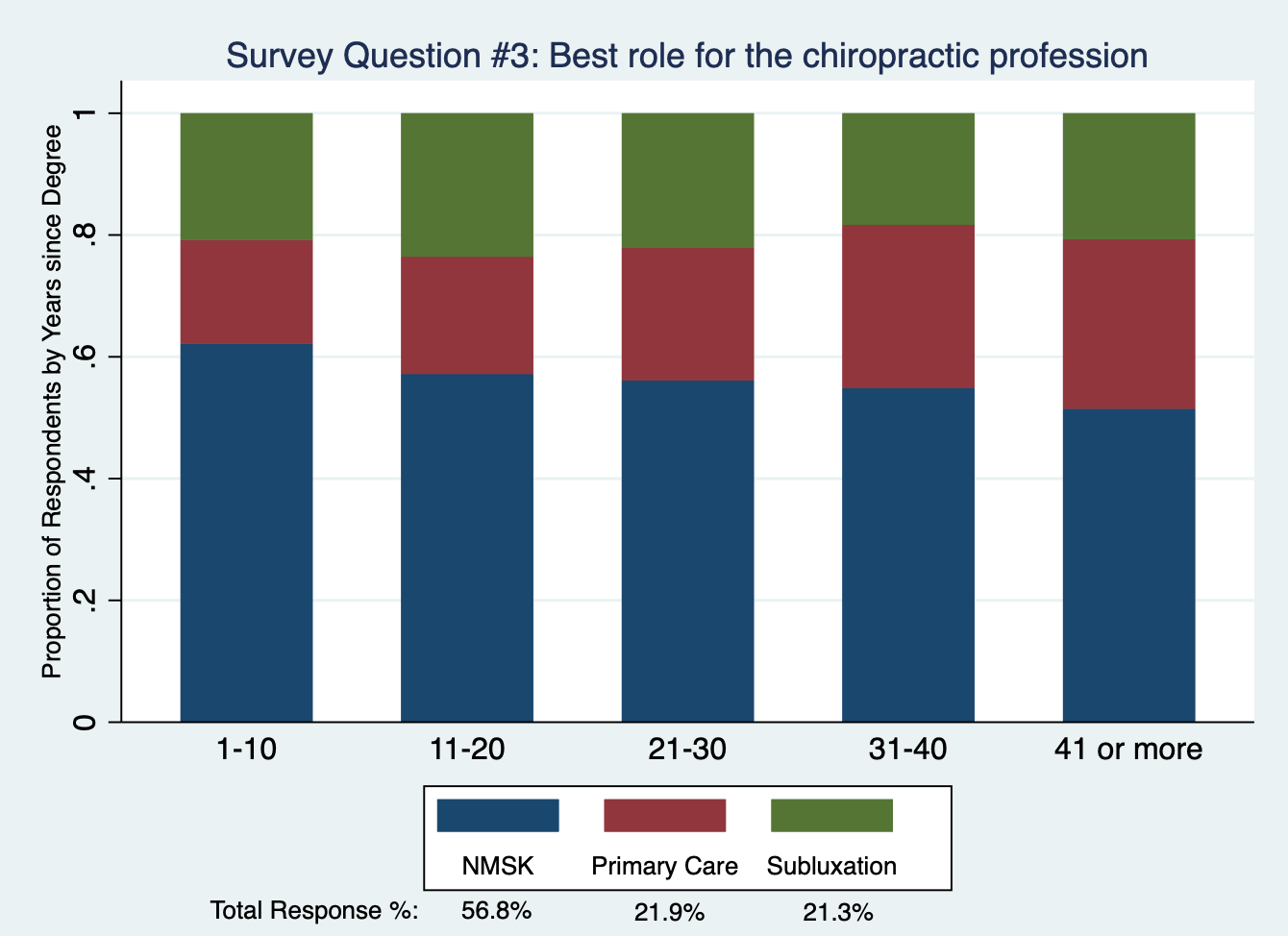 | 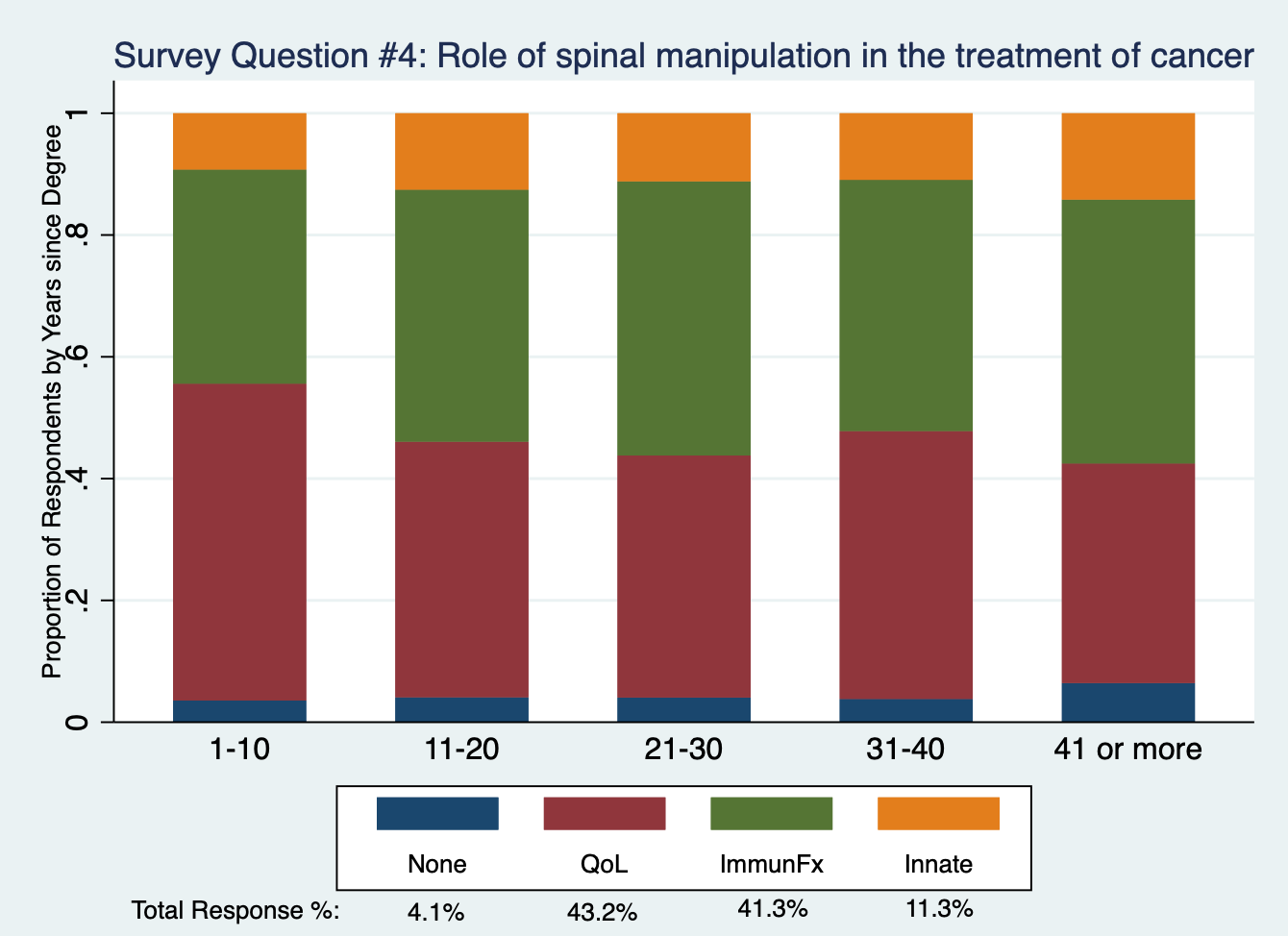 |
| 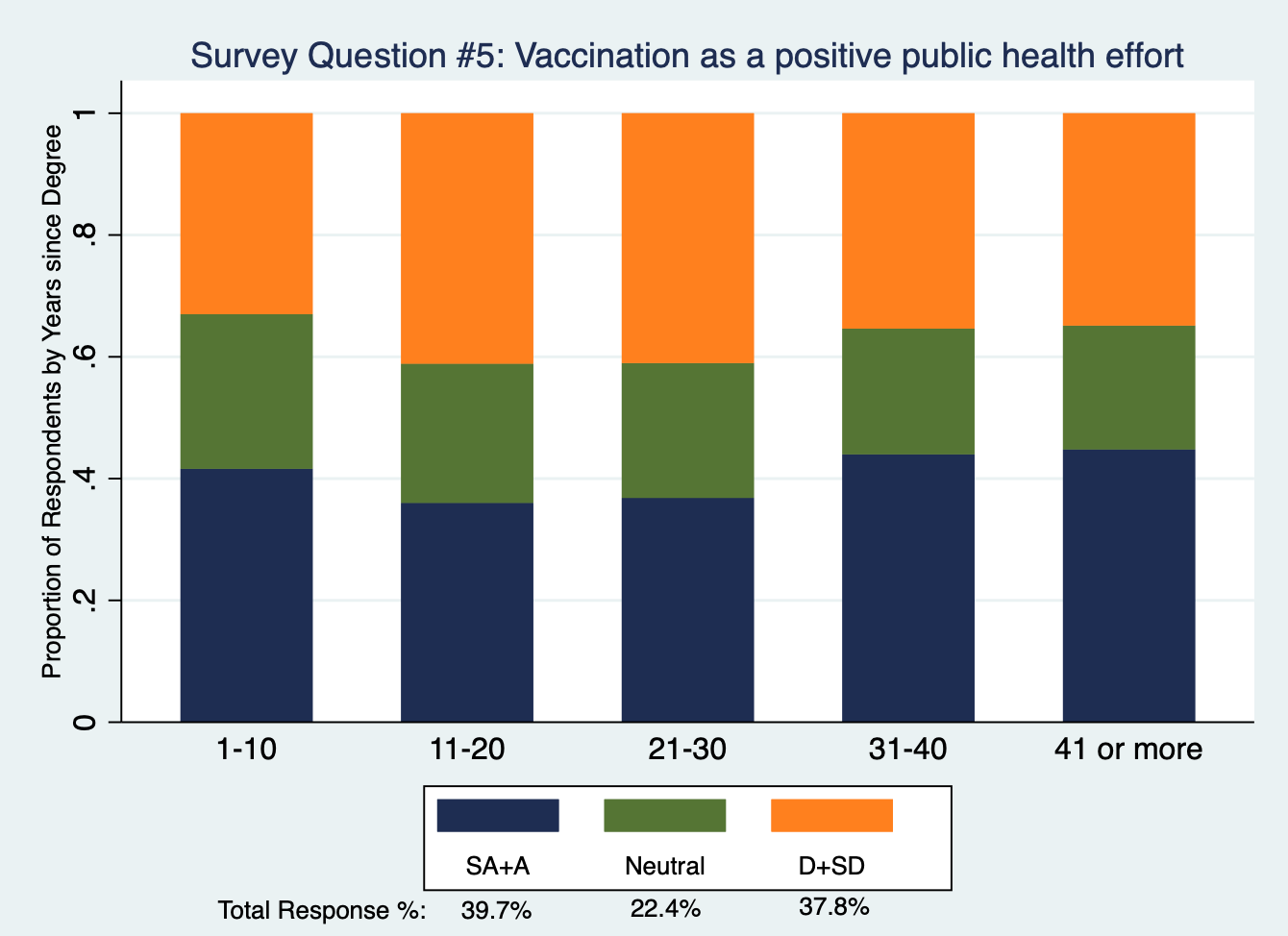 | 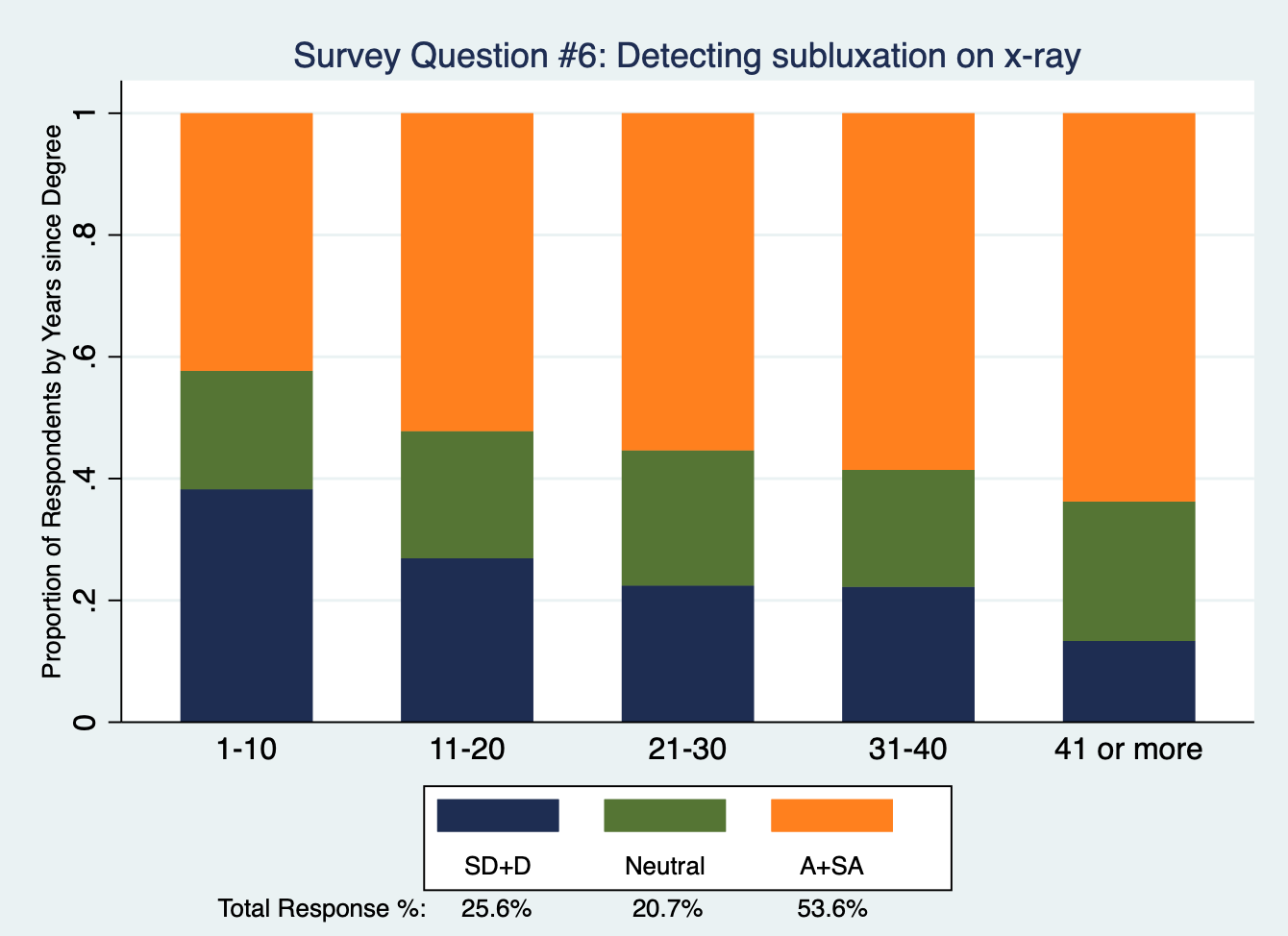 |
| 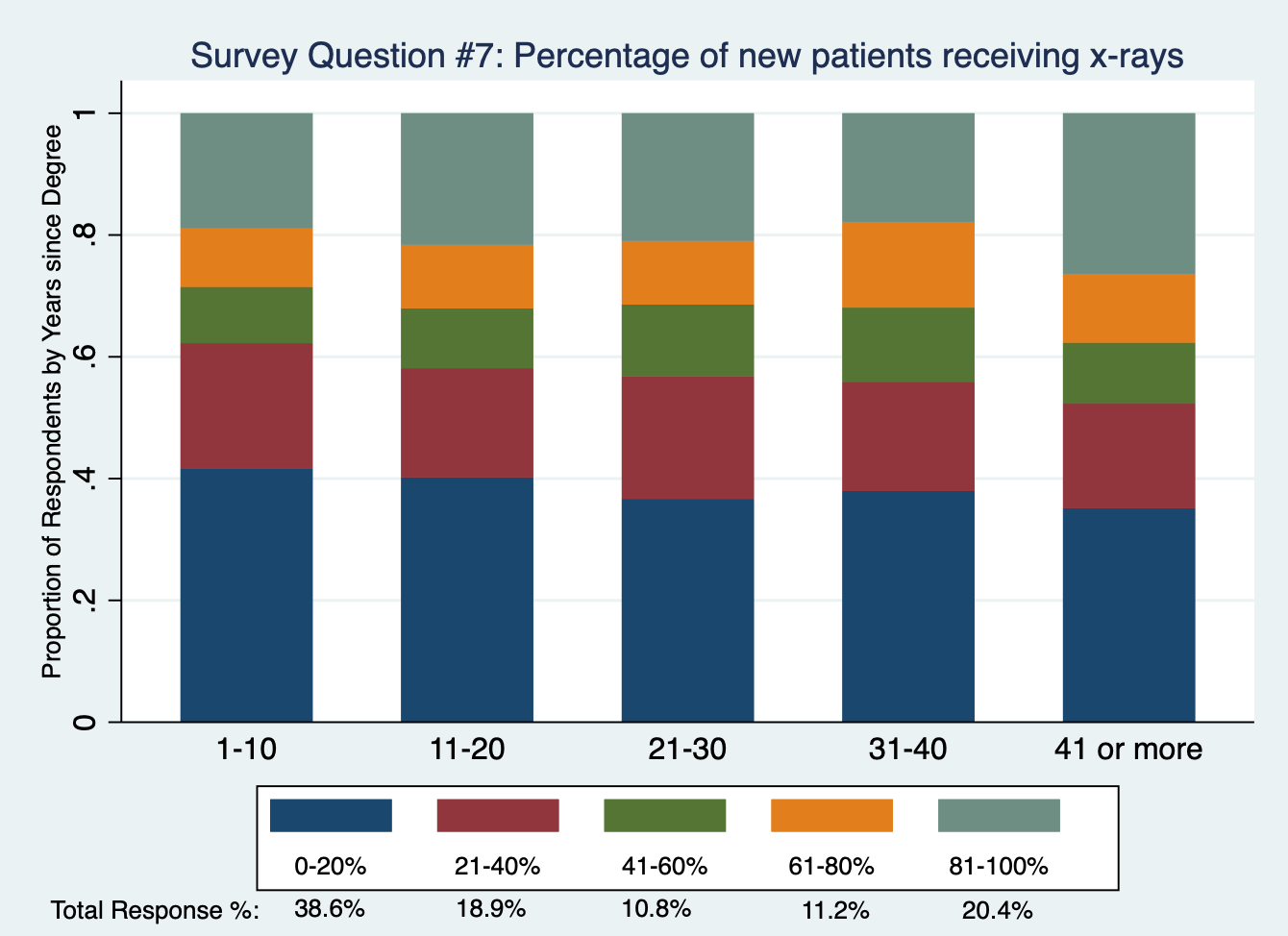 |  |

The figure legend is organized so that left to right corresponds to each stacked bar graph from bottom to top. In contrast to chiropractic degree program of graduation (Figure 2), the stacked bar graphs are sorted in chronological order left to right, from early career chiropractors (1-10 years), second decade of career (11-20), third decade of career (21-30 years), fourth decade and late career (31-40 years), and fifth decade on (41 or more years). The total response percentage reported below the figure legend represents the proportion of respondents for each answer from the entire survey sample.

Survey Question 1 labels: **DDx:** Differential Diagnosis only; **DDx>SA:** Focus on differential diagnosis, sometimes includes spinal analysis; **DDx+SA:** Equal focus on spinal analysis to detect subluxation and differential diagnosis; **SA>DDx:** Focus on Spinal analysis, sometimes includes differential diagnosis; **SA:** Spinal analysis to detect subluxation only

Survey Question 2 labels: **nMSK:** Neuromusculoskeletal Conditions; **MSKgen:** General and Biomechanical Conditions; **MSKsub:** Vertebral Subluxation as a Musculoskeletal Condition; **Somatovisc:** Biomechanical and Organic/Visceral Conditions; **Broad:** Broad Spectrum of Health Concerns Including Lifestyle and Wellness Issues; **VS:** Vertebral Subluxation as an Encumbrance to Health

Survey Question 3 labels: **NMSK:** spine and neuromusculoskeletal focused subgroup; **Primary Care:** General primary care focused subgroup; **Subluxation:** Subluxation detection and removal subgroup

Survey Question 4 labels: **None:** No Role; **QoL:** Improving Pain/Quality of Life; **ImmuneFx:** Improving Nervous System/Immune System Function; **Innate:** Removing Interference to Innate Intelligence

Survey Question #5 labels: **SA+A**: Strongly Agree and Agree responses; **SD+D**: Strongly Disagree and Disagree responses

Survey Question #6 labels: **SD+D**: Strongly Disagree and Disagree responses**; A+SA**: Agree and Strongly Agree responses
